# Pronounced methane cycling in northern lakes coincided with a rapid rise in atmospheric CH_4_ during the last deglacial warming

**DOI:** 10.1126/sciadv.adt2561

**Published:** 2025-07-16

**Authors:** Xinwei Yan, Jianbao Liu, Wengang Kang, Xianyu Huang, Aifeng Zhou, Lin Chen, Jifeng Zhang, Haoran Dong, Zhitong Chen, Junjie Wu, Henry Holmstrand, Kathleen M. Rühland, John P. Smol, Fahu Chen, Örjan Gustafsson

**Affiliations:** ^1^ Group of Alpine Paleoecology and Human Adaptation (ALPHA), State Key Laboratory of Tibetan Plateau Earth System, Resources and Environment (TPESRE), Institute of Tibetan Plateau Research, Chinese Academy of Sciences, Beijing 100101, China.; ^2^ College of Urban and Environmental Sciences, Peking University, Beijing 100871, China.; ^3^ Department of Environmental Science and the Bolin Centre for Climate Research, Stockholm University, Stockholm 10691, Sweden.; ^4^ Key Laboratory of Western China’s Environmental Systems (Ministry of Education), College of Earth and Environmental Sciences, Lanzhou University, Lanzhou 730000, China.; ^5^ State Key Laboratory of Biogeology and Environmental Geology, China University of Geosciences, Wuhan 430074, China.; ^6^ Research Center for Ecology, College of Science, Tibet University, Lhasa 850000, China.; ^7^ Key Laboratory for Geographical Process Analysis and Simulation of Hubei Province, College of Urban and Environmental Sciences, Central China Normal University, Wuhan 430079, China.; ^8^ Paleoecological Environmental Assessment and Research Lab (PEARL), Department of Biology, Queen's University, Kingston, Ontario K7L 3N6, Canada.

## Abstract

Atmospheric methane concentration (AMC) surged by ~50% during the last deglaciation, with northern (>30°N) sources accounting for ~40% of the rise. However, hypothesized sources including expanding lakes, peatlands, and destabilized permafrost or hydrates fail to explain this rapid increase. We use biomarkers, isotopes, and radiocarbon data to reconstruct temperature change, methane cycling, and permafrost thaw from a Tibetan thermokarst lake. Radiocarbon evidence and ultradepleted δ^13^C values (−80.3 per mil) of methane-diagnostic lipids indicate intense cycling of ancient (~2500-year-old) methane during the Younger Dryas–Preboreal transition, coeval with the AMC surge and the most rapid warming. By contrast, methane cycling was weak during the Holocene Climatic Optimum despite peak temperatures. These findings imply that anomalously high rates of warming, rather than absolute temperature alone, may play an important role in triggering enhanced paleo-methane cycling. Rapid warming likely intensified emissions from existing northern lakes, fueling the elusive yet clearly amplified northern methane source that contributed to the deglacial abrupt rise in AMC.

## Teaser

Pronounced methane emissions from northern lakes in response to warming contribute to abrupt deglacial atmospheric CH_4_ rise.

## INTRODUCTION

Methane (CH_4_) is a potent greenhouse gas, with natural emissions accounting for about half of current total global emissions (*1*). However, large uncertainties remain regarding how these emissions respond to climate forcing, particularly from large climate-sensitive, old carbon reservoirs (*2*–*5*). Understanding the factors driving the rapid rise in atmospheric methane concentrations (AMC) during the last deglaciation can provide insights into these uncertainties and help anticipate methane-climate feedbacks under current and future warming scenarios. Atmospheric CH_4_ concentrations are predominantly reflective of many emission sources yet are also mediated by sinks of methane in the soils (oxidation) and atmosphere (reaction with the hydroxyl radical OH); changing source fluxes governed past abrupt CH_4_ changes (*6*, *7*). In addition to well-investigated tropical wetlands source (*8*, *9*), the differences in interpolar methane concentrations unambiguously indicate an increased contribution from northern (>30°N) regions during the last deglaciation, estimated to account for up to 45% of the additional budget to the global atmospheric methane (*10*–*13*).

The various sources of the northern contribution to the rapid rise in AMC during the Younger Dryas–Preboreal (YD-PB) transition have been intensely debated (*5*, *14*, *15*). Potential emissions from the dissociation of marine hydrates and thawing permafrost could have been precluded based on assumptions of source isotope signatures and interpretations of the isotope compositions (Δ^14^C and δD) of CH_4_ in ice cores (*2*, *16*). A distinct δ^13^CH_4_ enrichment in the Greenland ice cores was observed during the YD-PB period, suggesting a notable contribution from northern thermokarst lakes (*17*). However, basal age studies indicate that the rapid formation of northern lakes lagged the abrupt deglacial increases in AMC by ~500 to 1000 years; thereby, new-lake formation could not account for the Northern Hemisphere contribution to the abrupt AMC increase (*5*) nor could expansion of northern peatlands as these were formed even later (*14*, *18*). Although peatland precursors (e.g., shallow lakes and non-peat wetlands) and early successional stage peatlands (e.g., marshes and fens) are more notable CH_4_ sources (*18*), this may only partially offset the estimated temporal lag in methane emissions, it still falls short of explaining the abrupt increase in AMC. Yet, previous studies only considered an increase in the number of thermokarst and glacial lakes related to the ice sheet retreat and thawing of ice-rich permafrost ground; this is a relatively slow process and lags the initial warming signal by decades to centuries (*2*, *14*).

The above scenarios, however, do not take into account the potential for intensified methane cycling in existing lakes, which may respond quickly to rapid warming (*19*). Contemporary short-term experiments have shown methanogenesis is strictly and positively dependent on absolute temperature (*20*), and higher temperatures will strongly enhance freshwater CH_4_ emissions (*19*, *21*, *22*). This absolute temperature-methanogenesis paradigm has been used in paleo-methane emission modeling of northern lakes but has failed to identify an abrupt increase in methane emission during the last deglaciation (*5*, *18*). Ice cores from Greenland have demonstrated coeval and abrupt (decade-to-century) increases in Northern Hemisphere temperatures and AMC during the YD-PB abrupt warming event (*15*), whereas the vital role of the rate of temperature change in explaining the variability of AMC has not been carefully examined. Unfortunately, the investigation of long-term variations in lacustrine methane cycling—especially their magnitude and timing in relation to temperature changes (rate and absolute temperature)—has been hampered by a paucity of high-quality temporal records.

Permafrost soils on the Tibetan Plateau (TP) as well as in the circum-Arctic region store almost twice as much carbon as is currently present in the atmosphere (*23*). Thermokarst landscapes cover 20 to 40% of the global permafrost regions and store up to half its soil organic carbon (SOC) (*24*). In these systems, thermokarst lakes constitute the most widespread sites for abrupt permafrost thaw and provide a direct conduit for the processing and emission of permafrost carbon to the atmosphere in the form of CH_4_ and CO_2_ (*25*). Ebullition is the primary source of CH_4_ emissions of both TP and Arctic thermokarst lakes (*26*, *27*). Further to this rapid bubble-mediated transfer to the atmosphere, the characteristic low dissolved oxygen concentrations and shallow water columns of TP thermokarst lakes also allow for rapid diffusive flux, with very little time for CH_4_ removal by microbial oxidation (*26*). Hence, it is likely that a large fraction of the methane that is released to the lake waters is also emitted to the overlying atmosphere. Moreover, pre-aged (ancient) carbon released by thawing permafrost contains labile SOC capable of fueling methanogenesis (*25*, *28*). This has been partly deduced from the observation that the ^14^C age of methane in lakes is nearly identical to that of permafrost soil carbon that is thawing in their drainage basins (*25*). Thermokarst lake methane production is thus likely to increase with permafrost thawing, triggering a positive permafrost-CH_4_ feedback (*5*).

The last deglacial warming was found to trigger massive permafrost carbon remobilization (*29*–*31*). This provides the opportunity for evaluating the permafrost carbon dynamics and their influence on thermokarst lake methane cycling. To provide insights into the responses of methane activities in “pan-Arctic” (including circum-Arctic and TP regions; see Materials and Methods) thermokarst lakes and the possible release of ancient methane to climatic change, we investigated a detailed lake sedimentary archive from TP that extends back in time beyond the YD-PB transition. Indicators of paleoclimatic changes, permafrost thawing and variations in methane cycling were well expressed in this record and were representative of the typical response of thermokarst lake methane cycling to climatic change and permafrost thaw in the pan-Arctic domain.

**Fig. 1.**
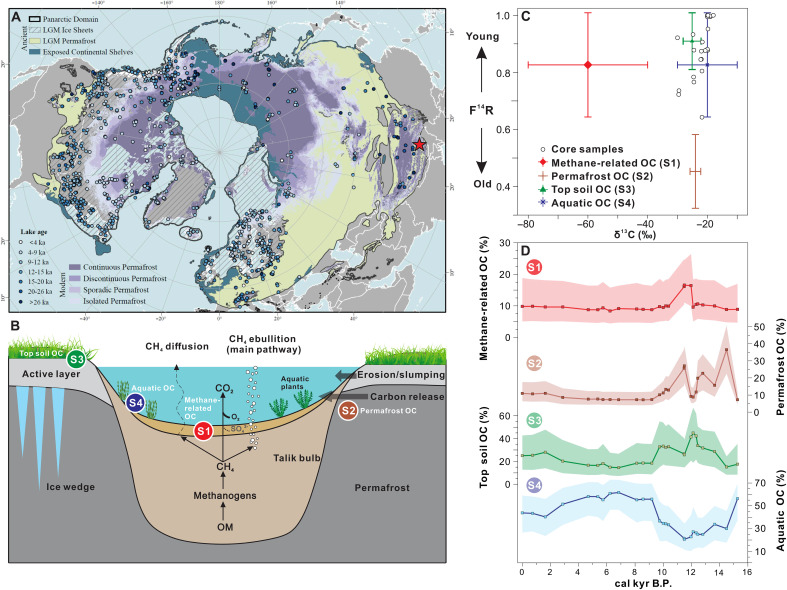
Pan-Arctic map, thermokarst lake carbon cycle, and sedimentary carbon source apportionments. (**A**) Locations of basal age records (circles color coded by age) within the pan-Arctic domain (black solid line) (*5*). The red star represents the studied thermokarst lake. Also shown is the distribution of modern permafrost zones (*23*). (**B**) Schematic diagram of a thermokarst lake microbial carbon cycle (*87*), illustrating how the microbial community within the unfrozen ground layer (Talik) degrades OM to CH_4_. The CH_4_ can escape via diffusion and ebullition (the dominant pathway, bypassing CH_4_ oxidation) (*27*). The four different carbon sources are depicted by S1 to S4. (**C**) BOC δ^13^C and F^14^R values of sediment samples (Materials and Methods; *n* = 26): The data points include only those BOC δ^13^C samples that have corresponding F^14^R measurements (white dots) and the range of endmembers for methane-related OC (red; S1), permafrost carbon (brown; S2), topsoil carbon (green; S3), aquatic OC (blue; S4) and used in a dual-isotope mixing model. (**D**) Trends in OC fractions from different carbon sources from sediment core (NBC19/21A) over time. The shading shows the interquartile range predicted by source apportionment. kyr, thousand years.

**Fig. 2.**
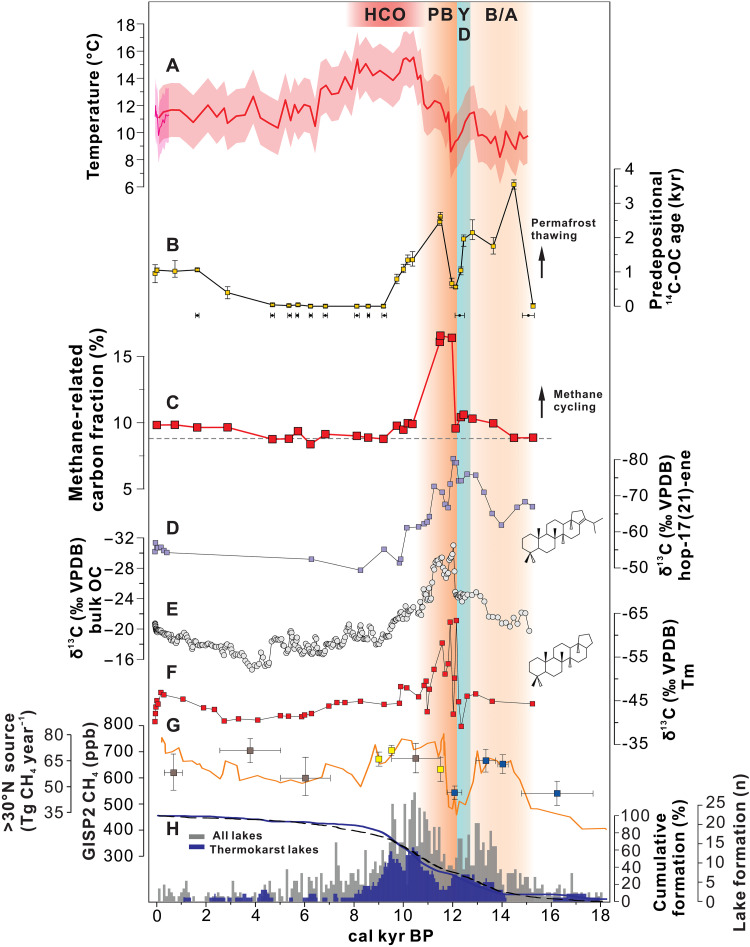
Coupling between changes in temperature, permafrost thawing, and methane cycling in Nianbu Co thermokarst lake, atmospheric methane changes, and the development of northern lakes over time. (**A**) MWST variation based on sedimentary brGDGTs from Nianbu Co short and long cores; the red shading shows the RMSE uncertainty envelope of the calibration, ±2°C (*42*). (**B**) Predepositional ^14^C age of OC; below are the age-control points. (**C**) Temporal changes of the methane-related OC fraction; the gray horizontal line represents the mid-Holocene background level. (**D**) hop-17(21)-ene δ^13^C values (structure shown). (**E**) BOC δ^13^C values. (**F**) Tm hopanoids δ^13^C values (structure shown). (**G**) AMCs from the GISP2 ice core (orange) (*15*); also shown are the discrete northern (>30°N) source methane contributions (means ± SD) inferred from the interpolar gradient by Dällenbach *et al.* (*13*) (gray), Baumgartner *et al.* (*10*) (blue), and Yang *et al.* (*12*) (yellow). (**H**) Basal ages of thermokarst lakes used in thermokarst-lake methane emission estimates (dark blue) and lakes used to model pan-Arctic lake methane emissions from the full suite of lake types (gray) (*5*). Solid (thermokarst) and dashed (all lake types) lines show the cumulative formation of lakes in each dataset as a fraction of the total. Vertical shading highlights the various climatic periods of this study including the HCO, the warm phases of the B/A and the PB, and the cold phase of the YD. ppb, parts per billion.

The thermokarst lake Nianbu Co [29°48′N, 92°22′E; 4980 m above sea level (a.s.l.)] is located in the Southern TP (Fig. 1A and fig. S1) (see Supplementary Text 1), a region that is sensitive to both Northern Hemisphere temperatures (Materials and Methods) and Indian summer monsoon (ISM) hydrological changes (fig. S2). The small catchment (<1 km^2^) facilitates rapid source-to-sink transfer processes, and organic matter (OM) “aging” and degradation during transport is negligible. We measured radiocarbon, biomarkers, and compound-specific isotopes from well-dated lake sediment cores (Materials and Methods; figs. S3 and S4) that include the recent anthropogenic warming period, the Holocene Climatic Optimum (HCO), and the last deglacial warming period. Using the depositional ages, based on radiocarbon dating of 10 terrestrial macrofossils (table S1) as well as ^210^Pb and ^137^Cs dating, radiocarbon contents of bulk organic carbon (BOC) were decay corrected to derive initial radiocarbon content and corresponding “predepositional ages” (Materials and Methods; Fig. 2B). Compound-specific isotope analysis (CSIA) was applied on deuterium/hydrogen of leaf-wax long-chain *n*-alkanes (δD_wax_) to reconstruct ISM intensity and, by extension, humidity changes (Materials and Methods; figs. S1B and S3). CSIA of the specific methanotrophy hopanoids δ^13^C was used to trace changes in aerobic methane oxidation and, by extension, methane cycling changes (Materials and Methods; Fig. 2, C and E). Branched glycerol dialkyl glycerol tetraethers (brGDGTs) were used to reconstruct warm-season temperature changes over time (Materials and Methods; Fig. 2A). After ruling out the presence of petrogenic carbon (^14^C-free), which may obscure the signature of permafrost OC (Materials and Methods), source apportionment of BOC through dual carbon isotope (δ^13^C and Δ^14^C) analyses were used to constrain temporal changes in OC fractions from different carbon sources (Materials and Methods; Fig. 1, C and D). This was combined with CSIA of hopanoids and the constrained predepositional ages in the sediment core to facilitate the pinpointing of past variations in permafrost thawing, export, and methane cycling.

## RESULTS

### Diagnostic lipid biomarkers linked to methane cycling

Methanotrophs function as lacustrine methane filters by oxidizing CH_4_ to CO_2_ (Fig. 1B), and their biomolecules provide a record of methane cycling that is preserved in sediment profiles. Methane is oxidized by both aerobic bacteria and anaerobic prokaryotes that can use a suite of alternative electron acceptors. Aerobic methanotrophy, performed by methanotrophic bacteria, is considered the primary CH_4_ filter in freshwater lakes (Fig. 1B).

Depleted carbon isotope composition (δ^13^C) of specific lipid biomarkers of methane-oxidizing bacteria (MOB) have been tightly linked to methane cycling, with these “fossilized methane” signals providing direct evidence for the extent of ancient methane release (*32*–*37*). For example, bacterial aerobic methane oxidation is linked to hopanoids such as diploptene and diplopterol (*32*, *33*), which are synthesized by microorganisms performing assimilative methanotrophy and are thus especially ^13^C depleted, with reported δ^13^C values ranging from −40 to −80 per mil (‰) (*32*–*37*). A strong correlation has been shown between methanotroph-specific hopanoid δ^13^C values and methane production (*38*). Here, we used CSIA measurements of hop-17(21)-ene and 17α(*H*)-22,29,30-trisnorhopane (Tm) (Fig. 2, C and E), the diagenetic product of diploptene and diplopterol (*39*), to reconstruct historical methane cycling. The similar trends in δ^13^C values of hopanoids and BOC in the core (Fig. 2, C to E) suggest strong methanotrophic activity and associated high production of ^13^C-depleted carbon in the lake. MOB can oxidize methane as an energy source, converting it into cellular matter or carbon dioxide gas, providing an important source of carbon in lake food webs and primary production (*40*). The increased utilization of methane and recycling of ^13^C-depleted CO_2_ (the product of the methane oxidation), and greater numbers of consumers that feed on MOB in an aquatic ecosystem can affect the δ^13^C signatures of the in situ OM in the lake (*37*). Therefore, the fraction of methane-related (CH_4_-derived) carbon in the sediments is a reflection of the intensity of methane oxidation in the lake. To further refine our analysis, we constrained the fraction of C that has a methane origin (methane-related C) and referenced it to the sedimentary total organic carbon (TOC), using statistical source apportionment based on ∆^14^C-OC and δ^13^C-OC (Materials and Methods) to conduct a simplified quantitative assessment of lacustrine methane cycling (Fig. 2F). Methane production by methanogens (methanogenesis) and its oxidation by methanotrophs (methanotrophy) drive the biogenic methane cycle, with the balance between the two ultimately controlling the amount of CH_4_ released into the atmosphere. The hopanoids δ^13^C and methane-related carbon fraction presented in this study reflect the amount of CH_4_ being oxidized in the lake. Methanotrophy is known to have a more positive effect on substrate availability (i.e., CH_4_) than temperature, whereas methanogenesis is particularly sensitive to temperature (*19*). Thus, the inferred high rates of aerobic methane oxidation occurring in the water column are most likely related to elevated CH_4_ production in the sediments. Moreover, the buffering capacity of methanotrophy to methanogenesis in lakes is limited because of their different sensitivities to temperature, with a disproportionate increase in methanogenesis over methanotrophy when temperature increases (*19*). Collectively, it is reasonable to conclude that the amount of methane oxidation, as traced by these advanced molecular-isotopic “fossil methane” proxies, has a positive correlation with CH_4_ emissions from lakes. This relationship is particularly evident in thermokarst lakes, where ebullition serves as the dominant methane release pathway (*26*).

### Temperature changes since the last deglaciation

**Fig. 3.**
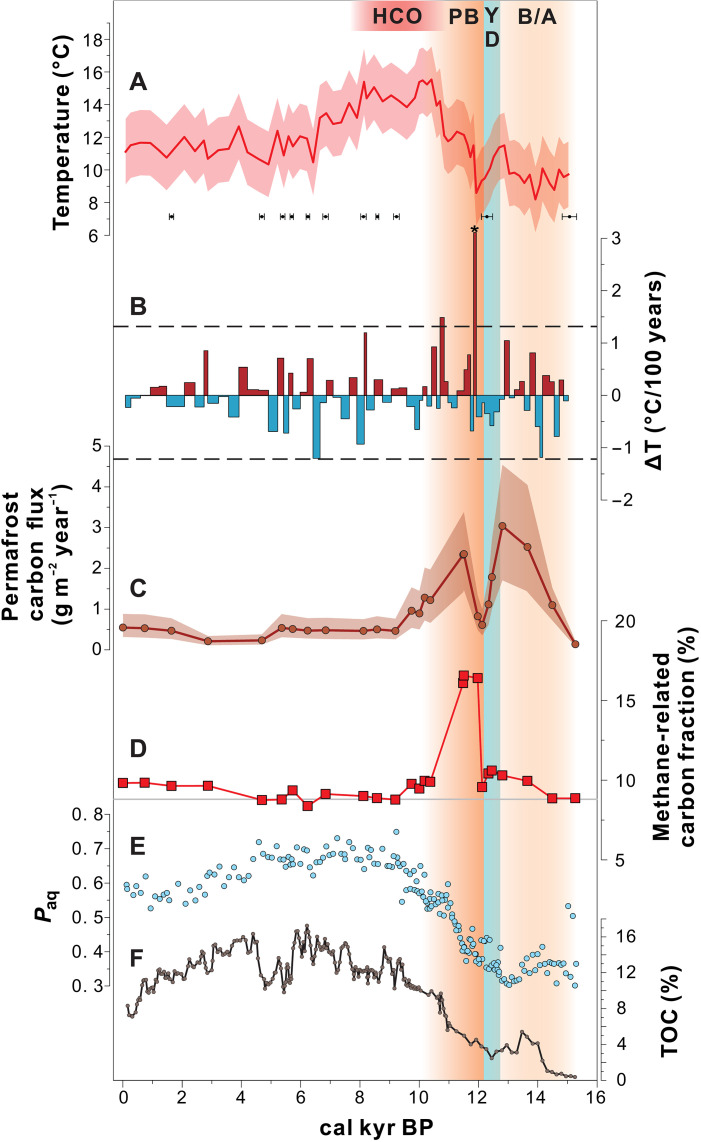
Intensity of methane cycling compared with the rate of temperature change, permafrost carbon input, and TOC content in Nianbu Co thermokarst lake over time. (**A**) Absolute temperature variation derived from brGDGTs (below are the age-control points) and (**B**) corresponding temporal variations in the rate of temperature change with the mean value ± 2 SDs (black dashed horizontal lines): Asterisk denotes the most rapid warming rate (3.6°C/100 years). (**C**) Permafrost carbon flux, calculated by multiplying the permafrost OC fraction by the TOC accumulation rate. (**D**) Methane-related OC fraction represents changes in the intensity of the methane cycling; gray horizontal line represents the mid-Holocene mean value (background level). (**E**) Lipid biomarkers of aquatic versus higher plants (*P*_aq_) and (**F**) sedimentary TOC content. Vertical shading highlights the various climatic periods of this study including the HCO, the warm phases of the B/A and the PB, and the cold phase of the YD.

**Fig. 4.**
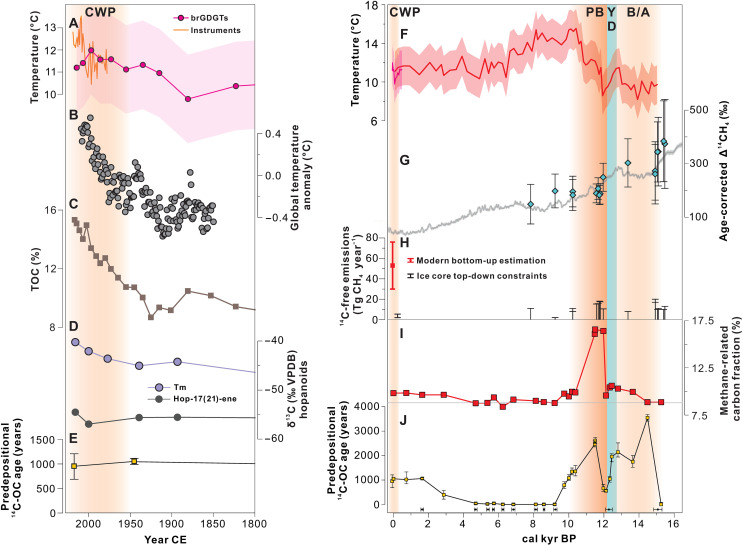
Proxy trends from the Nianbu Co sediment record showing the comparison of methane cycling and the permafrost carbon ^14^C age during the last deglacial warming and the CWP. (**A**) Trends in brGDGT-based temperature (magenta circles) from the Nianbu Co sediment record (the shading shows the uncertainty envelope of the calibration, ±2°C) and instrumental (orange solid line) MWSTs (April to August) from the Mozhugongka County weather station. (**B**) Global mean air temperature anomaly (*88*). (**C**) Sedimentary TOC. (**D**) δ^13^C values of hop-17(21)-ene and Tm. (**E**) Predepositional ^14^C age of recent Nianbu Co sediments. (**F**) Trends in brGDGT-based warm-season temperature (red) from the Nianbu Co sediment record (the shading shows the uncertainty envelope of the calibration, ±2°C). (**G**) Δ^14^CH_4_ retrieved from the Antarctic ice core and Δ^14^C of contemporaneous CO_2_ from IntCal13 (gray line) (*2*, *3*). (**H**) Global modern-day bottom-up geological CH_4_ (^14^C-free) emission estimates (red) (*56*) and top-down geological CH_4_ emission estimates (black) derived from the ice cores (*2*–*4*). (**I**) Trends in methane-related OC fraction from the Nianbu Co sediment record with the gray horizontal line representing the mid-Holocene mean value (background level). (**J**) Changes in predepositional ^14^C age of OC from Nianbu Co sediments over time; below are the age-control points. Vertical shading highlights the various climatic periods of this study, including the warm phases of the B/A, the PB, and the current warm period (CWP), and the cold phase of the YD.

Aquatic methane emissions are closely correlated with warm-season temperatures (*22*), which has also been supported by the observation that the highest atmospheric methane columnar concentrations occur in summer over the TP (*41*). brGDGTs (membrane lipids produced by bacteria) have been widely used as warm-season paleo-thermometers for cold region lakes (*42*). During the last deglaciation, our brGDGT-derived warm-season temperature reconstruction (Materials and Methods) shows moderate warming during the Bølling-Allerød (B/A) period, cooling during the YD, and abrupt warming at the transition period of the YD-PB (Fig. 2A), characterized by the fastest rate of warming (3.1°C/100 years) over the entire time span of the record (Fig. 3B). During the Holocene, the brGDGT-derived temperatures are consistent with a composite record of Northern Hemisphere temperature changes (*43*) (fig. S5), characterized by the highest temperature (~14.8°C) during the early Holocene [10.4 to 8 thousand years ago (ka)], i.e., the HCO (*43*). The temperature reconstruction from recent sediments exhibits a persistent warming trend after the end of the Little Ice Age, capturing the current warming period (CWP) (Fig. 4A). Notably, the reconstructed warm-season temperature falls within the range of instrumental values and uncertainties (Fig. 4A and fig. S5).

### Deglacial permafrost carbon remobilization on the TP

Abrupt permafrost carbon remobilization during the last glacial-interglacial transition has been observationally deduced in several sedimentary records. For example, several marine sediment core studies from the circum-Arctic have established large-scale enhanced release of terrestrial carbon, which, when combined with their ^13^C and ^14^C contents, point to source domains such as fluvial discharge from surficial inland permafrost (relatively younger) and from erosion of coastal permafrost (relatively older) (*29*–*31*). Such evidence for massive remobilization of previously frozen carbon with reported high biodegradability and hence lability to microbial decomposition (*25*, *28*), was likely associated with the simultaneous release of greenhouse gases into the atmosphere.

Our radiocarbon dating of BOC in the thermokarst lake sediment profile demonstrates similar patterns of pre-aged permafrost carbon remobilization in the Tibetan alpine permafrost region during the last deglaciation, suggesting a coherent hemispheric permafrost response to climate change. The predepositional ages of BOC were oldest (1750 to 3500 years) during deglaciation (15 to 10 ka) with a temporary decline to ~500 years at the YD event, when cold climate restabilized the permafrost (Fig. 2B). During deglaciation, two distinct peaks of predepositional ages of ~3500 and 2500 ^14^C years old correspond to B/A warming and YD-PB abrupt warming events, respectively (Fig. 2B). These Tibetan permafrost thawing events were coeval with and thus most likely caused by climatic warming as the brGDGT-derived temperature increased from 8.2° to 11.5°C and from 8.6° to 15.5°C during the B/A and YD-PB, respectively. A deglacial increase in thermal erosion is also confirmed by a microbial source indicator—the Methane Index (MI). The MI is a proxy linked to anaerobic oxidation of methane in marine environments, whereas in small lakes that are influenced by terrigenous input, the MI is related to catchment soil erosion (*44*). During the last deglaciation, the lower MI values as well as the higher mass accumulation rates in our sediment record indicate a higher erosion/slump activity and vulnerable permafrost conditions compared to the Holocene (fig. S6) (see Supplementary Text 2). These abrupt thaw periods may rapidly affect meters of permafrost, vastly increasing the carbon pool for decomposition and leading to high carbon emissions.

## DISCUSSION

### Amplified methane cycle triggered by a fast rate of warming rather than by high temperatures

It is noteworthy that the most ^13^C-depleted values of hop-17(21)-ene (~−80.3‰) and Tm (~−63.3‰) occurred during the YD-PB abrupt warming event, clearly signaling a methane origin that is coeval with the decline in bulk OC δ^13^C values (~−31.0‰) (Fig. 2). There is an obvious decrease in the Tm δ^13^C value of >20‰, from about −39 to −63‰ (Fig. 2F). This indicates that there was a large contribution of methanotrophs to the bacterial biomass carbon pool during the YD-PB transition. Enhanced methane consumption and recycling of methane-related products (^13^C-depleted) may have contributed to the large carbon isotope excursions recorded in the BOC, from about −25 to −31‰ (Fig. 2E). The fraction of methane-related carbon increased by ~75% from about 0.095 to 0.166 during the YD-PB transition (Fig. 2C), demonstrating unparalleled intensification of methane oxidation that persisted for ~1000 years.

Substrate (i.e., CH_4_) availability is known to have a more positive effect than temperature change on methanotrophy (*19*); hence, the intensified CH_4_ consumption identified by the carbon isotope excursions was probably a response to an increase in lacustrine CH_4_ production during this abrupt warming event. By directly comparing the reconstructed warm-season temperatures with the ^13^C-depleted hopanoids and the methane-related fractions, we found that the appearance of the largest carbon isotope excursions generally coincided with the most rapid rate of warming, i.e., 3.1°C/100 years, when the temperature increased from 8.6° to 12.3°C (Fig. 3). The shift in the hopanoid δ^13^C value is slightly preceding (i.e., ~100 to 200 years) the rapid increase in brGDGT-derived temperature (Fig. 2). This is likely because hopanoid compounds, being smaller, migrate downward through sediments more easily (*45*) than the larger, more structurally complex brGDGTs. The high polarity of brGDGTs results in strong binding with mineral surfaces, making them less mobile in sediment. Alternatively, the composition of brGDGTs might be influenced by changes under oxic conditions (*46*) during this exceptionally strong methane cycling period, which suppressed the response of brGDGTs to the initial warming signal. Nonetheless, the YD-PB warming event, lasting ~1000 years, aligns well with the periods of intensified methane cycling inferred from ^13^C-depleted hopanoids and increased methane-related carbon fractions, further supporting the strong association between methane cycling and climatic warming (Fig. 3).

It has been suggested that lacustrine methane cycling is strongly dependent on temperature, with higher temperatures leading to higher rates of methanogenesis (*22*). However, we find no evidence for an intensification of methane cycling during the later HCO, the period with the highest temperatures (Figs. 2 and 3). Instead, the largest perturbations to the freshwater methane cycling were exclusively limited to the YD-PB warming, as demonstrated by the highest methane-related fraction (Figs. 2 and 3). These observations suggest that absolute temperature is not the primary factor governing paleo-methane cycling. Rather, temperature-driven variations in ecosystem state (e.g., substrate availability and quality, microbial community structure and/or composition, and physiological acclimation and/or adaptation) and hydrological condition (e.g., changes in water level) play critical roles in modulating the response of methane cycling to climatic warming (*20*). Extreme warming events in the past are characterized by anomalously high rates of temperature increase, which can induce rapid shifts in hydrology and terrestrial, marine, and cryospheric ecosystem dynamics—often exhibiting nonlinear relationships with absolute temperature (*29*, *34*, *47*). Such abrupt shifts may interact synergistically with rising temperatures to trigger transient but intensified methane cycling. For instance, during the YD-PB transition, a transient yet intensified episode of permafrost thaw (Fig. 3C) likely altered substrate supply and quality by releasing microbially reactive carbon (*25*, *28*). When combined with rapid warming, this joint effect may have triggered intense methane cycling (Fig. 3). It should be noted that reactive permafrost carbon input alone can only slightly increase methane cycling, as demonstrated during the moderate warming of the B/A or the stable climate of the late Holocene (Fig. 3, C and D) and is further supported by the weak correlation (*R*^2^ = 0.19, *P* < 0.05) between the methane-related carbon fraction and the permafrost carbon fraction (fig. S8).

A decoupling between absolute temperature and the methane cycle in high-latitude wetlands has also been observed during the Paleocene-Eocene Thermal Maximum (PETM; a “hyperthermal” event at ~56 million years) (*33*, *34*). This major perturbation of the methane cycle appears to have been confined to the onset of the PETM and did not persist into the early Eocene, despite sustained elevated temperatures (*34*). Although direct evidence remains limited, several hypotheses have been proposed to explain this decoupling, including increased primary productivity, altered substrate supply, and an unstable hydrological regime during the onset of the PETM (*34*). Despite these events occurring on vastly different timescales, the similar patterns of methane cycling in response to warming point to an alternative mechanism: Extensive remobilization of permafrost carbon during the PETM (*48*) may have contributed to enhanced methane cycling in high-latitude wetlands under warming conditions. These observations suggest that transient but intense paleo-methane cycling is more strongly associated with the rate of climatic change than with an equilibrium response to sustained warming.

In addition, the variability of hopanoid δ^13^C values during the YD-PB transition in Nianbu Co thermokarst lake is larger than those in other depositional settings with active methane cycling yet comparable to that during the PETM perturbation. The most ^13^C-depleted sample (~−80‰) is even lower than those of PETM samples (albeit the specific hopanoids are different; fig. S7), suggesting highly vigorous methane cycling in the TP system during the YD-PB abrupt warming event.

In addition to temperature effects, the size and depth of small lakes in the ISM zone are prone to be influenced by the weakening of monsoons during the mid-late Holocene (see Supplementary Text 3), which, in turn, affects methane emissions (*49*). However, lake Nianbu Co, as an outflow lake, has maintained a relatively stable size and depth since its formation (see Supplementary Text 1). In addition, the trends in mass accumulation rate and sedimentation rate differ from monsoon precipitation, showing higher values during the deglaciation period (figs. S6 and S9), suggesting that the accumulation of detritus in the lake is predominantly regulated by permafrost thawing rather than runoff. Moreover, a previous study suggested that primary productivity may influence the rates of lacustrine methane emissions (*49*). Nonetheless, the aquatic OC fraction and the *P*_aq_ value (representing lipids ascribed to aquatic plants versus lipids derived from higher vascular plants) (*50*) and sedimentary TOC content (Fig. 3, E and F) suggest much higher primary productivity and carbon content during the warm middle Holocene than during deglaciation, but the intensity of methane cycling was opposite (Fig. 3D). Therefore, primary productivity and total carbon availability cannot account for the methane cycling dynamics recorded here.

Collectively, we therefore conclude that anomalously high rates of warming, rather than absolute temperature alone, may play a more important role in triggering enhanced paleo-methane cycling. Our results highlight the need for caution when identifying the driving factors of methane cycling in permafrost regions.

Methane flux changes across ecosystems have been linked strictly to absolute temperature, based on the Boltzmann function (*20*). Existing studies have used this relationship to estimate paleo-methane production (*5*, *18*). However, our comprehensive records suggest this temperature-methane paradigm (positive correlation) may not be universally applied across time periods. Our detailed analyses of lake records reveal that, in contrast to the abrupt warming event of the YD-PB, the highest temperatures of the HCO resulted in weakened methane cycling and likely a diminished contribution of lake methane emissions. Our current study finds that the marked spike in methane cycling during the YD-PB was triggered by a high rate of warming. This may be used to improve previous modeling efforts that simply parameterized apparent CH_4_ production and emissions as a static function of absolute temperature. These findings may thus also help to resolve mismatches in temperature data-models and could yield higher estimates of the northern source CH_4_ contribution across periods of abrupt rises in global AMC.

### Pan-Arctic lakes as an important contribution to atmospheric methane surge

During the YD-PB transition, there was a sharp increase in AMCs that coincided with rapid northern warming (*15*). This instantaneous response and synchronization are also evident in the Nianbu Co sediment record, with abrupt temperature increases and sudden intensification of methane cycling during this transitional period. In contrast to the sharp rise in temperature and AMC during the YD-PB transition, the moderately warm interval of the B/A had relatively weak reconstructed methane cycling in the lake record (Fig. 2C), which is consistent with the slower AMC increase. Therefore, prior to the YD-PB, the total methane emissions from northern lakes might have been primarily governed by the rate of lake formation (Fig. 2H).

Current paleo-methane emission modeling typically focuses on the formation rate of pan-Arctic lakes and peatlands (*5*, *14*) (fig. S10), which lagged the initial warming signal and a sharp rise in AMC of the YD-PB transition. This lag can be explained by the thermal inertia of the ice-rich permafrost system and by ice sheet retreat (*5*, *14*). A presumed link between absolute temperature and methane production has also been used in modeling simulations (*5*). Such model estimates of paleo-methane can only yield a gradual increase in northern methane emissions, thereby failing to capture the sharp increases in AMC during the YD-PB transition. It is noteworthy that more than 30% of the northern region’s thermokarst and other types of lakes had accumulated prior to the YD-PB transition (Fig. 2H). The transient but abrupt temperature rise during the YD-PB originated primarily in the North Atlantic with hemisphere-scale effects (*51*), which had the potential to cause instantaneous and considerable perturbations in the methane dynamics of pan-Arctic lakes. This may have contributed to the coeval rise in AMCs and mixing ratios in the Northern Hemisphere (Fig. 2G). The mechanism behind warming-driven CH_4_ emissions from northern lakes may differ from that of tropical CH_4_ emissions, where wetlands are the dominant source of methane (*9*) (fig. S11). In the tropics, deglacial CH_4_ emissions are instead largely a response to shifting rainfall patterns that expand wetland areas and increase microbial CH_4_ production (*8*). This is probably because, in the tropics, there are fewer lakes than in the northern extra-tropics, and the rate of warming at the onset of the Holocene in low-latitudes (tropic) was substantially slower than in mid- to high-latitude regions (*51*), making them more sensitive to changes in hydrology than temperature (fig. S11). The evidence for intensified CH_4_ cycling during the YD-PB in the TP record provides a useful analog for similar environments in the circum-Arctic region (i.e., low temperature with ice-cover and high warming rates), which likewise responded to YD-PB warming. The TP region is experiencing a warming rate similar to that of the Arctic, which is much faster than the global average (*52*). This suggests that both the TP and the Arctic exhibit high climate sensitivity. Given their similar environmental conditions, the characteristics of lake greenhouse gas emissions are likely comparable, with CH_4_ emission rates from TP lakes falling within the range observed in circum-Arctic lakes (*52*) and consisting predominantly of young carbon (*26*, *53*). The temperature-methane response mechanism of the YD-PB proposed in this study is probably universal and could be applied to various types of lakes in the pan-Arctic permafrost domain (Fig. 1A). Together, it is highly likely that CH_4_ cycling in circum-Arctic freshwater systems exhibits a similar response to warming as observed in this TP lake. Therefore, we conclude that highly intensified methane emissions from preexisting pan-Arctic water bodies, as a near-instantaneous response to warming, may have been an important northern CH_4_ source that contributed to the abrupt increases in AMC during deglacial periods.

Given that the fraction of methane-related carbon increased by ~75% (from 0.095 to 0.166) during the YD-PB transition, we make the assumption that lake methane emissions increased by at least the same level as increased temperatures would strongly enhance methane emissions through a disproportionate increase in methanogenesis over methanotrophy (*19*). Current estimates suggest that methane emissions from pan-Arctic lakes at the time of the AMC surge—at the end of the YD (~11.5 ka)—were ~15.5 [9.5 to 21.4] Tg CH_4_ year^−1^, with over half of this contribution (9.4 [5.9 to 12.9] Tg CH_4_ year^−1^) originating from thermokarst lakes and the remainder from glacial lakes (*5*). Taking into account the amplification factor of 1.75 derived from our study lake, we estimate that total methane emissions from pan-Arctic lakes could have reached 27.5 [17 to 38] Tg CH_4_ year^−1^ at the onset of YD-PB. It should be noted that this estimate likely represents an upper estimate as glacial lakes are less affected by permafrost thawing than thermokarst lakes (*5*) and likely exhibit a lower amplification factor. Nonetheless, this abrupt increase in lake methane emissions may account for up to half of the 60 ± 7 Tg CH_4_ year^−1^ of northern source emissions inferred from interpolar ice core gradients at the onset of YD-PB (*12*). The remaining northern CH_4_ budget may have been contributed by emissions from extra-tropical wetlands, which may respond similarly to YD-PB rapid warming (*33*, *34*). Notably, boreal–Arctic wetlands are estimated to emit 20.3 ± 0.9 Tg CH_4_ year^−1^ under current warming conditions (*54*). It should be recognized that these are approximate estimates that rely on several assumptions, such as lake methane emissions are scaled proportionally with methane-related carbon, and that the amplification factor derived from this TP lake is, on average, representative of other pan-Arctic lakes. This assumption carries uncertainties due to the heterogeneity of CH_4_ emissions across pan-Arctic lakes (*5*, *22*, *26*), making the exact average amplification factor inherently unknown. In addition, this scaling estimate depends on the accuracy of previous baseline estimates of pan-Arctic lake methane emissions (*5*). Despite these limitations, this represents, to our knowledge, the first estimate of pulsed methane emissions from northern lakes during the YD-PB transition. Although we are unable to robustly constrain the CH_4_ contributions from pan-Arctic lakes to the YD-PB methane budget, our results indicate a rapid amplification of lake methane emissions during the YD-PB transition, which may partially explain why AMCs rise abruptly, rather than gradually, as is proposed by models (*5*). Hence, the proposed forcing mechanism offers an opportunity to bridge these model-data gaps during the last deglaciation and other AMC surge events, such as Dansgaard-Oeschger (D-O) events (see Supplementary Text 4).

### Ancient methane emissions triggered by the last deglacial warming

The likelihood of climate warming triggering the rapid release of CH_4_ trapped in high-latitude permafrost is currently debated. Both top-down and bottom-up approaches seem to have large uncertainties in estimates of geologic emissions (*55*). One recent top-down study, which used radiocarbon data of methane from Antarctic ice cores, suggested a limited contribution of methane from thawing permafrost during the abrupt YD-PB warming event (*2*, *3*). Fossil (^14^C-free) CH_4_ emissions have been estimated to range from 0 to 15.4 Tg CH_4_ year^−1^ during the YD-PB transition (*2*, *3*) (Fig. 4H). This flux estimate is much lower than present-day global bottom-up estimates of geological fossil (^14^C-free) CH_4_ emissions, ranging from 30 to 76 Tg CH_4_ year^−1^ (Fig. 4H), which have also been verified by various top-down estimates (*56*).

The relatively low emission estimate of fossil CH_4_ for the last deglaciation assumes a greater amount of stable old carbon reservoirs (such as permafrost) during the rapid YD-PB warming, which is greater than the current rapid warming period. In addition, this estimate of low natural geological CH_4_ emission agrees well with other preindustrial estimates based on ice cores (1.9 to 5.4 Tg CH_4_ year^−1^) (*4*) (Fig. 4H), suggesting a relatively stable old carbon reservoir during both the last deglaciation and during the Holocene. Collectively, this indirect method indicates that current anthropogenic warming may not trigger future large releases of aged CH_4_ from ^14^C-depleted carbon sources, such as old permafrost systems (*2*, *3*). However, the ice core data do not allow for a direct measurement of the radiocarbon content of methane emitted from permafrost systems during the past periods and, as such, the assumptions underlying this conclusion may require further scrutiny. By assuming an average age of permafrost-derived CH_4_, the ice core data can provide further valuable insights into the emission budget derived from old CH_4_ in permafrost (*2*); however, the large uncertainty surrounding the permafrost carbon age limits our understanding of the stability of ancient carbon reservoirs—particularly permafrost—during the last deglacial warming periods.

It has been demonstrated that the ^14^C age of methane emitted from thermokarst lakes is identical to the age of the thawing permafrost soil carbon in the catchment (*25*). We assume that this relationship can be extrapolated to periods in the past; thus, by comparing the methane cycling (indicated by the methane-related carbon fraction) and corresponding permafrost soil carbon age (indicated by predepositional age), we can evaluate the long-term stability of methane emission from permafrost system under a changing climate. We found an intensive ~2500-year-old methane cycling period during the YD-PB transition and a moderate ~2000- to 3500-year-old methane cycling period during the B/A warming (Fig. 4, I and J). These intensified methane cycling processes are generally indicative of increased methane emissions from lakes (*36*, *37*). Notably, the deglacial methane emissions are older and stronger than the present-day ~1000-year-old methane emissions, which is consistent with previous findings that modern methane emissions from TP thermokarst lakes are primarily fueled by young carbon decomposition (*26*). These observations suggest greater instability and remobilization of somewhat older carbon reservoirs during warm deglacial periods (Fig. 4, I and J). The results from the current study also suggest that the age of methane derived from thawing TP permafrost systems, including thermokarst lakes, during the last deglaciation was likely younger than circum-Arctic permafrost systems (*2*). Hence, additional similar reconstructions from a variety of sites across much broader permafrost regions are required to better constrain the input data used for source apportionment calculation of old carbon reservoirs.

Although phases of the HCO were likely warmer than today (*57*), the complicated temperature-methane interactions make this a less suitable analogy for future projections of pan-Arctic lake methane emissions. Given that the CWP is characterized by particularly high magnitude and accelerated rates of warming due to anthropogenic emissions, the pronounced spike in lake methane cycling during the rapid warming of the YD-PB transition may be the best analog for modern environments and for predicting future climate scenarios. Collectively, our results suggest that an acceleration of methane release from climate-sensitive pre-aged permafrost carbon sources may be anticipated with ongoing anthropogenic warming.

## MATERIALS AND METHODS

### Site description and sampling

The pan-Arctic region was here defined as all areas that are affected by the Last Glacial Maximum (LGM) permafrost extent (including the circum-Arctic and the TP region), the LGM ice sheets, and exposed continental shelves during the LGM through time (Fig. 1A) (*5*). Nianbu Co is a small (0.07 km^2^) thermokarst lake located on the southeastern TP (29°48′N, 92°22′E; 4980 m a.s.l.), on the southern border of the pan-Arctic region (Fig. 1A). The mean annual air temperature (MAAT) of the study site is ~−0.2°C (assuming a lapse rate of 0.6°C per 100 m) and mean annual precipitation (MAP) is ~560 mm (fig. S2). On the basis of field observations, the modern lake is shallow with a maximum water depth of ~2 m, with a flat lakebed and abundant submerged plants. Seasonal runoff enters the lake on its southern shore with an overflow to the north, suggesting a relatively short hydraulic retention time and stable lake depth and surface area (Fig. 1C and fig. S1). A comprehensive description of the lake setting is provided in Supplementary Text 1 and Shen *et al.* (*58*).

Six surface soil samples were collected within the small catchment (<1 km^2^). A 71-cm surface core (NBC19-GC-2) and a longer sediment core (NBC19A; 5 m) were retrieved from the center of the lake in September 2019 using a Universal gravity corer and a Livingstone piston corer, respectively. A 7-m-long sediment core NBC21A was obtained at the same position of the lake in March 2021 (fig. S3). The short core (NBC19-GC-2) was sectioned on-site using a close-interval extruder into 0.5-cm intervals, and the long cores (NBC19A and NBC21A) were subsampled at 1-cm intervals. All subsamples were freeze-dried. A reconstructed full profile, labeled as NBC19/21A, included the NBC19A and NBC21A cores, covering the past ~15,000 years of sediment accumulation.

### Sediment chronology

Surface sediments from Nianbu Co were dated using ^210^Pb, ^137^Cs, and ^226^Ra measured by direct gamma counting of 20 samples from the upper 20 cm of the surface core, using a high-purity well-type germanium detector (ORTEC GWL-120-15) at the State Key Laboratory of Tibetan Plateau Earth System, Resources and Environment, Institute of Tibetan Plateau Research, Chinese Academy of Sciences. Each sample was freeze-dried and ground to pass through a 100-μm mesh. Samples were weighed before and after the drying to calculate bulk densities and estimate mass depth. Dry sediment (~3 g) was packed into a polyethylene tube and sealed with Parafilm for 3 weeks to allow equilibration between in situ ^226^Ra and ^214^Pb, the latter measured as a proxy for supported ^210^Pb. Efficiency calibration and autoabsorption corrections were performed using a reference standard [^210^Pb, ^137^Cs, and ^226^Ra (via ^214^Pb)] from the China Institute of Atomic Energy. Each sample was measured for 22.2 hours (80,000 s). Unsupported ^210^Pb activity was calculated by subtracting ^226^Ra activity from total ^210^Pb activity for each interval. The ^210^Pb chronology of the core was determined using the constant rate of supply (CRS) model (*59*), which accounts for variation in sediment accumulation rates. The CRS model was further constrained with the chronologic marker of peak fallout from nuclear weapons testing in approximately 1963 (^137^Cs). The upper 20-cm sediment covers the past ~170 years based on the CRS model.

For the NBC19/21A long cores, radiocarbon ages were determined using an accelerator mass spectrometry device (AMS ^14^C) and were conducted on 10 samples of terrestrial plant macrofossils as well as one BOC sample at the bottom of the core. The BOC sample represents sediments that were deposited during the cold glacial period (with lowest TOC content being <0.4%), and it is assumed that the corresponding reservoir age offset is 0. The radiocarbon dates were calibrated using OxCal 4.4 (*60*) and the IntCal 20 calibration curve (*61*) and reported with 2σ age ranges, expressed as calendar years before CE 1950 (cal B.P.). An age-depth model of NBC19/21A was then developed using the “rbacon” package in R (Bayesian Accumulation Model, version 3.1.1) (*62*). The Bacon age-depth modeling approach uses prior information about regional accumulation rates with estimates of the rate of change of accumulation rates to estimate an accumulation rate at each sample depth using a gamma autoregressive process. Collectively, the ^14^C data used to establish the Bayesian age-depth model come from 10 terrestrial plant remains and one BOC, ensuring that the age-model is unaffected by the ^14^C reservoir effect and robust. Moreover, one radiocarbon date derived from terrestrial plant remains provided a date of ~12.2 ka (around the YD period), which falls clearly within the range of uncertainty of the Bayesian age-depth model output, further validating the robustness of the age-model during the last deglaciation. The constraint of the changes in hopanoids and BOC δ^13^C to the YD-PB (onset of the Holocene) transition is further corroborated by the reconstructed temperature and precipitation changes. The YD-PB transition is characterized by rapid increases in temperature and precipitation (*63*), which are well represented by changes in the brGDGTs and δD_wax_ proxies registered in this core and align with the timing of the negative excursion in BOC δ^13^C (Fig. 2 and fig. S6). On the basis of these lines of evidence, the most likely timing for the intensification of methane cycling was during the YD-PB period.

### Stable isotope ratio analysis and OM composition

Approximately 0.3 mg of dried sample powder was packed into a tin capsule for the C and ^13^C/^12^C analysis. Total contents of carbon and the bulk organic δ^13^C values were measured using an elemental analyzer coupled with an isotope ratio mass spectrometer (IRMS) (Vario Isotope Cube-Isoprime, Elementar) at the Third Institute of Oceanography, Ministry of Natural Resources, China. The sample was combusted at 950°C in a combustion tube, and the reduction of gases occurred at 600°C reduction tube with the carrier gas flow (He) of 200 mL min^−1^. The δ^13^C values were calibrated with the certified reference material acetanilide#1, which had a δ^13^C value of −26.85‰ relative to the Vienna Pee Dee belemnite (VPDB) standard. The precision of the δ^13^C analyses is <0.20‰.

### Lipid extraction and CSIA

All of the subsamples were freeze-dried, homogenized, and ultrasonically agitated four times (15 min each) in dichloromethane:methanol (v/v = 9:1) to ensure that all extractable lipids were retrieved. The total lipid extract was separated over a silica gel using hexane, dichloromethane, and methanol in sequence as eluents to the apolar (containing nonpolar hydrocarbons of *n*-alkane and hopanoids lipids) and the polar fractions (containing GDGTs), respectively. The polar fractions of these samples were redissolved and filtered through a 0.45-μm polytetrafluoroethylene (PTFE) filter prior to analysis. To enable subsequent δ^13^C analysis of the hopanoids and δD of long-chain *n*-alkanes (δD_wax_), urea adduction was used to separate cyclic (i.e. non-adduct, containing hopanoid lipids) and aliphatic (i.e. adduct, normal and isoalkanes) hydrocarbons. To achieve this, 0.5 ml of hexane, 0.7 ml of acetone, and 2 ml of urea (10% in MeOH) were successively added to the apolar fraction. The mixture was then frozen for 12 hours until urea crystals formed. The solvent was then collected as the cyclic fraction. The urea crystals were then dissolved in 0.5 ml of water, and the aliphatic fraction was extracted (×5) with ~1 ml of *n*-hexane.

The aliphatic fractions (*n*-alkanes) were measured using an Agilent 8890 gas chromatography (GC) unit equipped with a flame ionization detector (FID) and a split-injector, DB-5 GC column (30 m by 0.32 mm by 0.25 μm), with an external C8-C30 standard for quantification. The detailed oven program was as follows: hold at 40°C for 1 min, heat to 150°C at 10°C/min, ramp (heat) to 310°C at 6°C/min, and hold at 310°C for 10 min. The polar fractions (GDGTs) were performed using an Agilent 1290 series ultraperformance liquid chromatography–atmospheric pressure chemical ionization–6465B triple quadrupole mass spectrometry (UPLC-APCI-MS) system. When the flow rate was 0.2 ml/min, an aliquot of 15 μl was injected and separated on three Hypersil GOLD Silica columns in sequence (each 100 mm by 2.1 mm by 1.9 μm, Thermo Fisher Scientific, USA), maintained at 40°C. GDGTs were eluted isocratically with 84% A and 16% B for the first 5 min, where A = *n*-hexane and B = EtOA, followed by a linear gradient change to 82% A and 18% B from 5 to 65 min, then changed to 100% B for 15 min, and then back to 84% A and 16% B to equilibrate the pressure. Analyses were performed using the selective ion monitoring (SIM) mode. The analyses were carried out in the Key Laboratory of Western China’s Environmental Systems, Lanzhou University. The annual average composition in precipitation of hydrogen isotopes (δD_precip_) in the study area has a value of −118‰ (*64*), whereas the uppermost sediment sample from the study lake has a δD_wax_ value of −226‰, suggesting a modern apparent isotopic fractionation (ε_a_) of −108‰ between precipitation (δD_precip_) and δD_wax_ due to plant vital effects. This value falls within the previously reported range (−112 ± 18‰; 1σ) identified on the TP (*65*), confirming the reliability of our measurements.

The identification of hopanoids was based on a comparison of retention times and mass spectra with published data (*36*, *66*, *67*). In our samples, we detected 10 hopanoids (fig. S12), but we focus on hop-17(21)-ene and Tm, which are well separated from other compounds. δ^13^C values of individual hopanoids [i.e., hop-17(21)-ene and Tm] were determined followed the program of Huang *et al.* (*66*) using a Delta XP isotope ratio mass spectrometer (Thermo Fisher Scientific) coupled to an Thermo Scientific TRACE GC with an Agilent DB-5MS column (30 m by 0.25 mm by 0.25 μm). Instrumental performance was verified by running an in-house *n*-alkane mixture (containing *n*-C_23_, C_25_, C_27_, C_29_, C_31_, and C_33_ alkanes), which was measured in duplicate between every six runs. Squalane (δ^13^C value: −19.8‰) was added as an internal standard for both samples and standards. The concentrations of hopanoids were based on androstane, assuming the relative response factor of 1. The analyses were performed in the State Key Laboratory of Biogeology and Environmental Geology, China University of Geosciences, Wuhan. Total ion chromatography was used for peak area integration, the characteristic ions for hop-17(21)-ene included mass/charge ratio (*m/z*) 231, 367, 395, and 410 and *m/z* 149, 191, 355, and 370 for Tm in our samples. Compound-specific δD analysis was performed on *n*-alkanes by GC isotope-ratio monitoring mass spectrometry (GCirMS) in the Department of Environmental Science and Analytical Chemistry, Stockholm University. The δD values were calibrated against saturated high-molecular-weight (HMW) *n*-alkanes using the reference substance mix A7 (Biogeochemical Laboratories, Indiana University). For details on the method, we refer to Vonk *et al.* (*68*). Duplicate analyses were used to confirm that the SDs of the carbon and hydrogen isotope determinations were better than ±0.5 and 5‰, respectively. The δ^13^C and δ^2^H values are reported in the delta notation (‰) relative to VPDB and Vienna standard mean ocean water (VSMOW), respectively.

### Radiocarbon analysis of bulk OC and plants

Seventeen BOC samples of the lake sediments were pretreated using an acid wash procedure. Briefly, 1 to 2 g of samples was weighed and reacted with 0.5 M HCl at 60°C; the acid was replaced daily until the solution became clear and then washed to neutral and oven-dried at 60°C. The samples were graphitized using the Auto Graphitization Equipment (AGE III) and measured using a compact AMS, Mini Carbon Dating System (MICADAS, IonPlus AG) in the Radiocarbon Laboratory of the MOE Key Laboratory of Western China’s Environmental System (Ministry of Education) of Lanzhou University. In addition, another 10 BOC samples were prepared and dated by Beta Analytic (USA) (*n* = 6) and the Tandem Laboratory University of Uppsala (Sweden) (*n* = 4). All terrestrial plant macrofossils (*n* = 10) and two submerged plant samples were prepared and dated by Beta Analytic (USA), following acid-base-acid procedures (tables S1 and S2).

### Predepositional ages of OC

Sample ^14^C contents are given in the nondimensional “F^14^C” notation, a measure of the radiocarbon content of a sample, and can thus be related to the radiocarbon (uncalibrated) age of the sample as follows (*69*)

C14age=−8033×ln(FC14)

To determine the amount of “pre-aging” that OM has undergone before burying in thermokarst lake sediment, we use the “reservoir offset” metric, defined (*70*) as a measure of the radiocarbon age offset, in ^14^C years, between two contemporaneous carbon reservoirs (*69*). In our case, the reservoir offset metric is defined as the ratio of F^14^C values from two contemporaneous carbon reservoirs (*x* and *y*) at a given time of deposition in lake sediment. Relative enrichment is given by the notation F^14^C in dimensionless units, as follows (*69*)

FRx−y14=FCx14FCy14

The more commonly enriched reservoir (i.e., contemporaneous atmospheric F^14^C) is placed in the denominator, and the numerator is the F^14^C value of BOC in our case. It is thus a measure of the predepositional age (in ^14^C years) of OM at the time of its deposition. The predepositional age or reservoir age offset can be determined

$$R_{x-y}=-8033\times \text{ln}\left(F^{14}R_{x-y}\right)$$

To calculate reservoir age offset values through time, it is necessary to “uncalibrate” BOC deposition ages derived from the core-age models. We apply to BOC ages (*n* = 16) the radcal script of the ResAge package (*71*), designed for pairs of a reservoir-derived age (^14^C age of BOC) and a corresponding weakly a priori known (with some uncertainty) calendar age (based on core-age models), following the method of Hein *et al.* (*69*). The program uncalibrates age-model-derived deposition ages, that is, it converts these to ^14^C years based on atmospheric calibration curve [from IntCal20 (*61*)] and calculates reservoir offset and F^14^R values for each input. Other samples (*n* = 10), which have both ^14^C ages of BOC (reservoir-derived ^14^C age) and terrestrial plant macrofossil (atmospheric ^14^C age) embedded in the same sediment layer, were applied using the rad2 script of the ResAge package for direct intercomparisons. For the uppermost sample (3.5 cm to 4 cm, year approximately 2017 CE), which contains nuclear bomb–derived radiocarbon, we obtained the atmospheric ^14^C content (Δ^14^C = −46~+18‰) from the atmospheric measurements in 2018 CE (*72*).

### Influence of fossil OC contributions

To evaluate whether the higher BOC ages were caused by variable admixture of fossil, that is, petrogenic contributions, we calculated the carbon preference index (CPI) of *n*-alkanes

$$\begin{array}{c} \begin{array}{c} \;\text{CPI}\\ =0.5\times \left(C_{23}+C_{25}+C_{27}+C_{29}+C_{31}\right)/\left(C_{22}+C_{24}+C_{26}+C_{28}+C_{30}\right)+\\ \;(C_{23}+C_{25}+C_{27}+C_{29}+C_{31})/\left(C_{24}+C_{26}+C_{28}+C_{30}+C_{32}\right) \end{array} \end{array}$$

Terrestrial and aquatic plants produce long-chain *n*-alkanes with elevated CPI values, whereas thermally altered and extensively degraded OM contains *n*-alkanes with a CPI of ~1 (*73*). CPIs exhibit a negative correlation with their initial radiocarbon content (indicated by F^14^R; *R*^2^ = 0.50, *P* < 0.01; fig. S8); if fossil OC contributes to BOC, a positive correlation would be expected; thus, we rule out that the observed age variations reflect contributions of petrogenic material.

### Source apportionments of OC

The Stable Isotope Mixing Models (“Simmr” package in R) (*74*) was used to estimate the relative contributions of four major OC sources (i.e., Topsoil-OC, Permafrost-OC, Aquatic-OC, and Methane-related OC) to sediment samples through time (Fig. 1, C and D). Topsoil-OC includes the contemporaneous fixed carbon and active layer young carbon, with δ^13^C = −25.1 ± 3‰ and F^14^R = 0.905 ± 0.095 (0 to 1690 ^14^C age) (*75*, *76*). The range of δ^13^C values was based on the modern topsoil δ^13^C dataset of TP (*77*) and considers the changes in vegetation type in the past retrieved from fossil pollen of the same lake (*78*). Permafrost-OC contains ancient carbon from permafrost soil, which has a mean F^14^R = 0.453 ± 0.13, corresponding to ~4300 to 9100 ^14^C age (*79*) and δ^13^C = −24 ± 1.8‰ based on modern survey (*75*, *76*). The active layer and permafrost soil F^14^R endmember values are close to those used in the Arctic region (*80*). Aquatic-OC (autochthonous OM) represents submerged aquatic plants (including benthic mosses), which could conceivably assimilate ^14^C-depleted dissolved inorganic carbon (DIC) originating from decomposition of thawing permafrost and/or oxidation of methane produced from pre-aged permafrost OC. This is supported by the radiocarbon dating of present-day submerged plants with a 1120 ^14^C age (collected from the gravity core), which is identical to the current reservoir age offset within the dating uncertainties (table S2). Also, a date for oogonia (aquatic) (10,160 ± 30 ^14^C years) at a depth of 374 to 375 cm is nearly identical to the date of the corresponding BOC (9970 ± 30 ^14^C years) (table S2). This implies that the age of the DIC pool used by aquatic plants is the same as the lake reservoir age offset, which is primarily influenced by inputs of pre-aged permafrost carbon. The highest *P*_aq_ values during the middle Holocene, which indicate contributions of predominantly aquatic plants to the TOC, corresponded to a zero predepositional age (Fig. 4), which again supports that the DIC assimilated by aquatic plants did not contribute aged OC signatures to the bulk sediment age. Therefore, the F^14^R value of aquatic-OC was assigned a wide range from 0 to 3500 ^14^C age (F^14^R = 0.822 ± 0.178) as the largest predepositional age was about 3500 years during the entire investigated period. Microbial oxidation of ancient methane could also potentially supply ^13^C-depleted inorganic carbon to submerged plant photosynthesis. Stable carbon isotope values of submerged aquatic plants in present-day Arctic lakes and Holocene sediment show no indication of methane oxidation as a source of DIC in the lakes (*81*). In addition, the CO_2_ emitted from thermokarst lakes in TP was not ^13^C depleted (average: −13.4‰, *n* = 30) (*26*). Therefore, we exclude such processes and assign the δ^13^C of aquatic-OC range from ~−10 to −30‰, according to the published dataset (*82*). Methane-related OC, methanotrophic biomass carbon, defined as the biomass carbon and lipid biomarker synthesized by microorganisms performing assimilative methanotrophy, which is highly ^13^C depleted (*35*), ranged from −40 to −80‰ according to the CSIA of hopanoids. Similar to aquatic-OC, the ^14^C age of methane-related OC ranged from 0 to 3500 years (F^14^R = 0.822 ± 0.178) as the methanotrophs could potentially use different ages of methane as a carbon source. The OC fractions predicted by source apportionment were further confirmed by corresponding independent proxies (fig. S13). The uncertainty of OC fractions is represented with an interquartile range.

### GDGTs distribution and brGDGT-based temperature reconstructions

The ternary diagrams show that the relative distributions of both brGDGTs and isoprenoid GDGTs (isoGDGTs) in the surface sediments differ considerably from those in the catchment soils (fig. S12B). The sediment was dominated by hexamethylated brGDGTs (IIIa, IIIb and IIIc) and had less tetramethylated brGDGTs (Ia, Ib, and Ic) compared to soils. GDGT-0 was predominantly in the form of isoGDGT (>97%) in the sediment; GDGT-1, GDGT-2, GDGT-3, and the crenarchaeol were only found in detectable amounts in the sediment, whereas higher abundances were found in soils, especially crenarchaeol (fig. S14). The overwhelming predominance of GDGT-0 (fig. S11B) is expected in cold region lakes as the number of cyclopentane rings in GDGTs from surface sediments increases with increasing lake temperature (*83*). The absence of crenarchaeol, in addition to low concentrations of GDGT-1 compared to −3, is similar to Arctic lakes, indicating that methanogenic or methanotrophic Archaea are likely predominant sources of GDGT-0 (*83*).

The differences between the distributions of GDGTs in surface sediments and catchment soils suggest an autochthonous source for brGDGTs in lake sediments, which is similar to one other study of TP lakes (*84*). The distribution of sedimentary brGDGTs closely resembles those found in high-latitude cold region lakes (fig. S14). Therefore, we reconstructed past mean warm-season temperatures (MWSTs) using a calibration set (*42*), which we considered to be the best for capturing temperature variability through time (Eq. 1), where GDGTs in square brackets refer to fractional abundances

$$\begin{array}{c} \text{MWST}=20.9+98.1\times \left[\text{Ib}\right]\\ -12.0^{}\times (\left[\text{IIa}\right]+\left[{\text{IIa}}^{\prime }\right])-20.5^{}\times (\left[\text{IIIa}\right]+\left[{\text{IIIa}}^{\prime }\right]) \end{array}$$ (1)

This equation is based on the distribution of branched GDGTs in 85 lakes from the Scandinavian Arctic to Antarctica (including 73 lakes located in cold environments with latitudes above 50°). This global calibration has high accuracy and precision [*R*^2^ = 0.88, root mean square error (RMSE) = 2.0°C, root-mean-square error of prediction (RMSEP) = 2.1°C] and is not strongly influenced by pH, conductivity or water depth (*42*).

We also compared our reconstructions of past temperatures with other calibrations using the MBT/CBT (methylation of branched tetraether/cyclisation of branched tetraether) index (*85*, *86*), all of which showing similar trends in temperature change, whereas the MBT/CBT calibrations considerably underestimated temperatures when compared with the instrumental values (fig. S5).

## Supplementary Materials

### This PDF file includes:

Supplementary Text Figs. S1 to S15 Tables S1 and S2 References
