# Supplementary material for: Pronounced methane cycling in northern lakes coincided with a rapid rise in atmospheric CH4 during the last deglacial warming
Source: Sci Adv. 2025 Jul 16;11(29):eadt2561. doi: 10.1126/sciadv.adt2561 (PMC12266101; doi:10.1126/sciadv.adt2561)
Supplement: Supplementary file 1 — Supplementary Text Figs. S1 to S15 Tables S1 and S2 References [file sciadv.adt2561_sm.pdf]

Science Advances
AAAS

Supplementary Materials for

Pronounced methane cycling in northern lakes coincided with a rapid rise in atmospheric CH_4 during the last deglacial warming

Xinwei Yan *et al.*

Corresponding author: Jianbao Liu, jbliu@itpcas.ac.cn; Örjan Gustafsson, orjan.gustafsson@aces.su.se

Sci. Adv. **11**, eadt2561 (2025)
DOI: 10.1126/sciadv.adt2561

This PDF file includes:

Supplementary Text
Figs. S1 to S15
Tables S1 and S2
References

1. Lake Nianbu Co setting and sediment core descriptions

Lake Nianbu Co (29°48′N, 92°22′E; 4980 m a.s.l.) is located in the southern part of the Tibetan Plateau. This freshwater lake covers an area of approximately 0.07 km^2 and features a flat lakebed with a maximum water depth of about 2 m, underlain by C-rich substrate. The lake is fed by several seasonal inflows on its southern side and has an outflow on its northern side (fig. S1). Based on mass accumulation rates (MAR) of sediment cores (i.e., the trends in MAR are continuous and without abrupt shifts) (fig. S9), the lake has experienced continuous sedimentation over the past ~15,000 yr, indicating that its water level and area have remained relatively stable, with water depth unlikely to have exceeded 2–3 m. The lakebed is densely covered with submerged aquatic macrophytes, with different types of vegetation observed near the shoreline and in the central part of the lake. The lake's diatom assemblages are dominated by benthic and epiphytic taxa. A frost mound is visible along the shoreline. Google satellite imagery reveals the presence of ice bubbles during the ice-covered period, indicating the lake as a hotspot for greenhouse gas emissions.

In terms of vegetation, lake Nianbu Co lies on the ecotone between forest and meadow. The vegetation surrounding the lake consists predominantly of alpine meadows, with plants such as *Salix, Rhododendron*, and *Potentilla* occurring in more sheltered areas, while *Artemisia* is more common in drier and more exposed zones. There are no trees in the vicinity of the lake, although forests are present at lower elevations to the east (*58*).

The primary data for this study were obtained from 7-m-long sediment cores retrieved from the center of the lake using a Livingstone piston corer. The sediment cores primarily consist of gray-black clay, rich in organic matter, with no evidence of bioturbation. The sedimentary process is continuous and uninterrupted, with laminations appearing at the base of the core (fig. S3).

2. Tibetan permafrost dynamics during the Holocene

It is noteworthy that TP permafrost was rather stable from ca. 9 to 4 ka, given a near zero pre-depositional age throughout the mid-Holocene, during which the warm-season temperatures were much higher than during the last deglaciation (Fig. 2). We attribute the stable permafrost conditions to be likely linked to the buildup of soil organic layers (soil organic horizons <0.3 m thick) during the Holocene Climatic Optimum (HCO). Soil organic horizons and peat can buffer the response of permafrost to climate change via thermal insulation effects, which can reduce active layer thickness, limit thermal erosion, and restrict the release of aged OC from permafrost soils (*89*). Although sedimentary proxies cannot provide direct information about the state of catchment soils, the lower mass accumulation rate (MAR) during the HCO (fig. S6), when monsoon rainfall reached its maximum since the last deglaciation, suggests well-vegetated and stable catchment soils. Peatlands are not currently present in the watershed, while surface soil organic layers are widespread across the modern landscape of the study area. It is possible that soil organic horizons accumulated during the warm and humid HCO, but the development of peat at such high altitudes (~4980 m a.s.l.) is less likely. A secondary factor contributing to the reduction in pre-depositional age could be that the study lake has an outlet, thus increased monsoon precipitation may strongly enhance the water turnover rate, facilitating the removal of pre-aged carbon from the lake water. Nonetheless, our records suggest that, during the HCO, a climate period with favorable hydrothermal conditions, vegetation development and the accumulation of soil organic carbon may have enhanced permafrost stability. Such a scenario is consistent with the observations of a weak negative correlation between δD_{wax} and BOC $F^{14}R$ (Methods) values ($R^2 = 0.43$, $p < 0.01$, fig. S8).

The pre-depositional age increases and remains at ~1000 yr during the late Holocene (ca. 0-3 ka) and into the current warming period (Fig. 4), which is consistent with that the modern TP and Arctic thermokarst lakes where CH_4 emission are fueled primarily by young carbon decomposition (*26*, *53*). Empirical models (*90*) based on global data compilations predict catchment soil ^{14}C ages of ~100 years in the present-day climate (i.e., mean annual air temperature (MAAT) = 6.4 °C, mean annual precipitation (MAP) = 560 mm; according to climate metrics measured at the Mozhugongka station, fig. S2), much lower than the ca. 1000 yr ^{14}C age observed here. Therefore, permafrost thawing and carbon release must be considered.

The dual-isotope source apportionment shows an increased contribution in top soil carbon during the late-Holocene, which suggests that the aged OC was mainly derived from the active layer during this period (Fig. 1D). In addition, human activities, such as livestock grazing, can also cause anomalously old radiocarbon ages of BOC (*91*). However, fecal sterols and stanols, indicators for humans or their grazing animals, were below the detection limits in nearby Paru Lake (~2.9 km), suggesting minimal anthropogenic influence throughout the Holocene (*92*). Wildfires were recently considered a driver of permafrost thawing (*93*); however, the minimal variation in combustion proxies (polycyclic aromatic hydrocarbons) during the middle to late Holocene (*92*) exclude such a scenario. As such, we suggest temperature and vegetation dynamics dominated the permafrost carbon thawing process in the TP.

3. *Hydroclimate changes since the last deglaciation*

Hydroclimate changes were reconstructed from long-chain *n*-alkane (leaf wax) hydrogen isotopes (δD_{wax}) (*94*) in the Nianbu Co sediment record and were based on the average of isotope fingerprints in the C-27, C-29, and C-31 *n*-alkanes, which are mainly terrestrial compounds (Methods). The modern apparent hydrogen isotopic fractionation (ε_a) in the study area is -108‰ (Methods). Trends in the sedimentary δD_{wax} generally followed the reported Indian Summer Monsoon (ISM) dynamics derived from speleothem $\delta^{18}O$ (*95*) and other proxies (fig. S15). As previously shown, lacustrine δD_{wax} in the study area reflects integrated, synoptic-scale ISM dynamics (*94*) and, by extension, local rainfall (fig. S2). The monsoon climate system of the study area is further characterized by the coupling of GDGT-based temperature and δD_{wax}-based humidity since the last deglaciation ($R^2 = 0.59$, $P < 0.01$; fig. S8). The δD_{wax} values dramatically decreased from -175 to -262‰ during the last deglaciation, indicating a climate transition from dry to wet associated with deglacial warming, with a reversal at YD cold spell. The minimum δD_{wax} values occurred between ca. 10 and 8 ka, suggesting the wettest climate occurred during the Holocene Climatic Optimum. The ISM strength gradually decreased since ca. 8 ka following the decreasing summer temperature and insolation intensity, while the large variability of δD_{wax} during the late-Holocene (0-3 ka) suggests a decoupling between temperature and humidity. The late-Holocene hydrological reversal (decoupling) is also recorded in other ISM proxies, despite differences in timing and magnitude (fig. S15), suggesting that the underlying mechanism may have change (*96*).

4. *Implications for other abrupt atmospheric CH_4 rise events*

The abrupt warming events that coincided with equally abrupt increases in atmospheric CH_4 rise have been widely observed during the geological record. For example, during the last glacial period, Greenland experienced a series of abrupt temperature increases ranging from 8 °C to 16 °C within just a few decades. These abrupt warming events, known as Dansgaard-Oeschger (D-O) events, are also reflected in ice-core CH_4 records, showing CH_4 increases of up to two-thirds of the

glacial-interglacial concentration change (or up to 200 ppbv.) within just a few decades (*97*). However, none of the Last Glacial Maximum model simulations can reproduce the 100–200 ppbv increase required to match these observations (*97*). The hypothesis of a tropical methane source driving the D-O events is unsupported by the Paleoclimate Modelling Intercomparison Project (PMIP) simulations. Simulations using two wetland models of varying complexity indicate that methane emissions from tropical wetlands alone are insufficient to account for the D-O events (*98*). This suggests that either key wetland processes are missing in the models or that additional methane sources are necessary to explain these events (*98*). A recent study suggests that these AMC pulses are linked to increased pyrogenic CH_4 emissions caused by shifts in tropical rainfall (*7*). Nonetheless, the warming rate-related mechanism proposed and lake methane intensity spike found in this study may enhance extra-tropical methane contributions and improve model simulations, potentially reproducing the methane pulses required during D-O events.

Supplementary Figures

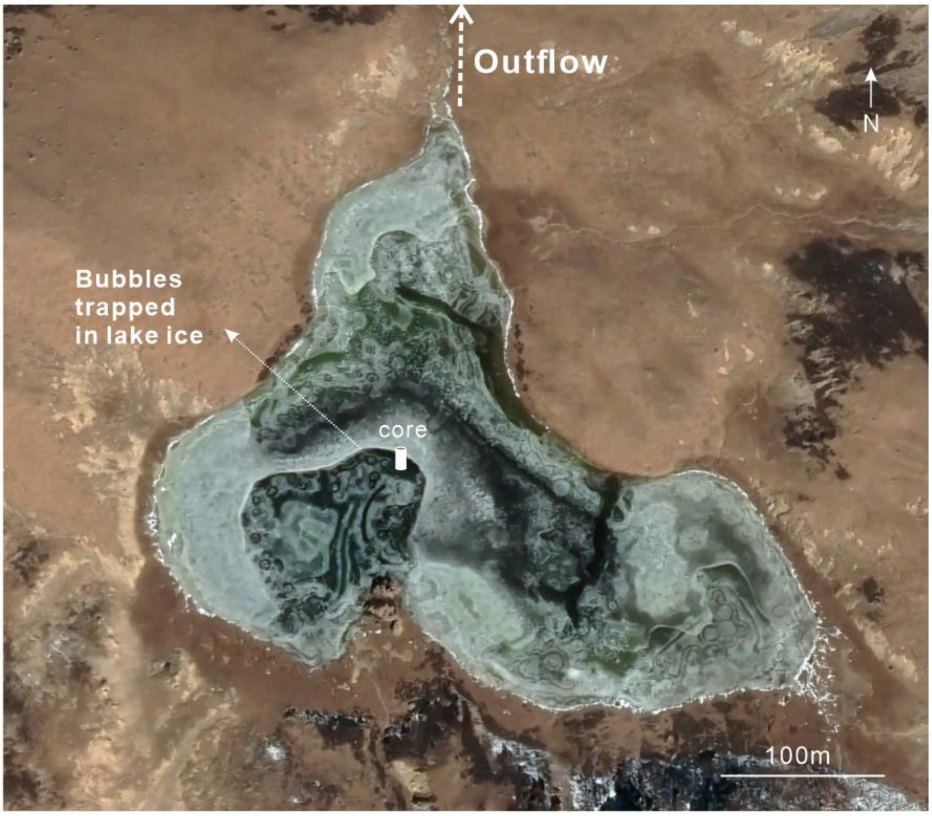

Fig. S1. Lake Nianbu Co setting and location of the sediment core. The lake has an outflow on its northern side. Google satellite imagery reveals the presence of ice bubbles during the ice-cover period, indicating the lake as a hotspot for greenhouse gas emissions.

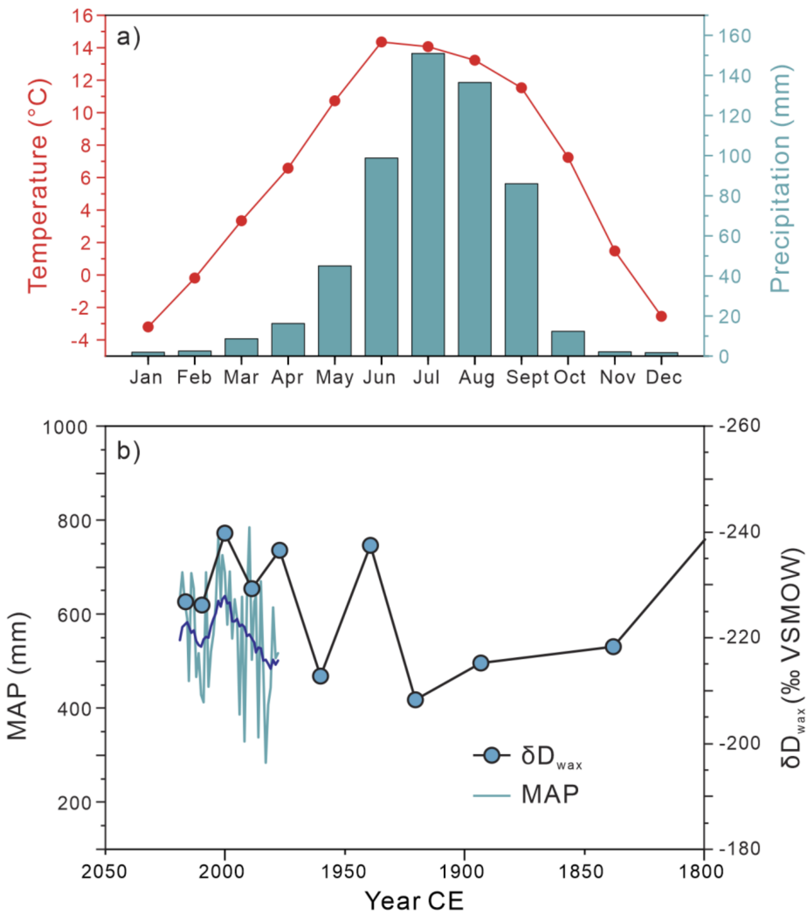

Fig. S2. Instrumental climate records and δD$_{wax}$ changes. a) Average monthly precipitation and air temperatures based on data from the Mozhugongka County weather station (~60 km from Nianbu Co) from 1978 to 2019. **b**) Comparison of instrumental mean annual precipitation (MAP) with δD$_{wax}$ measured from surface sediments; the dark blue line represents the 10-year moving average of MAP.

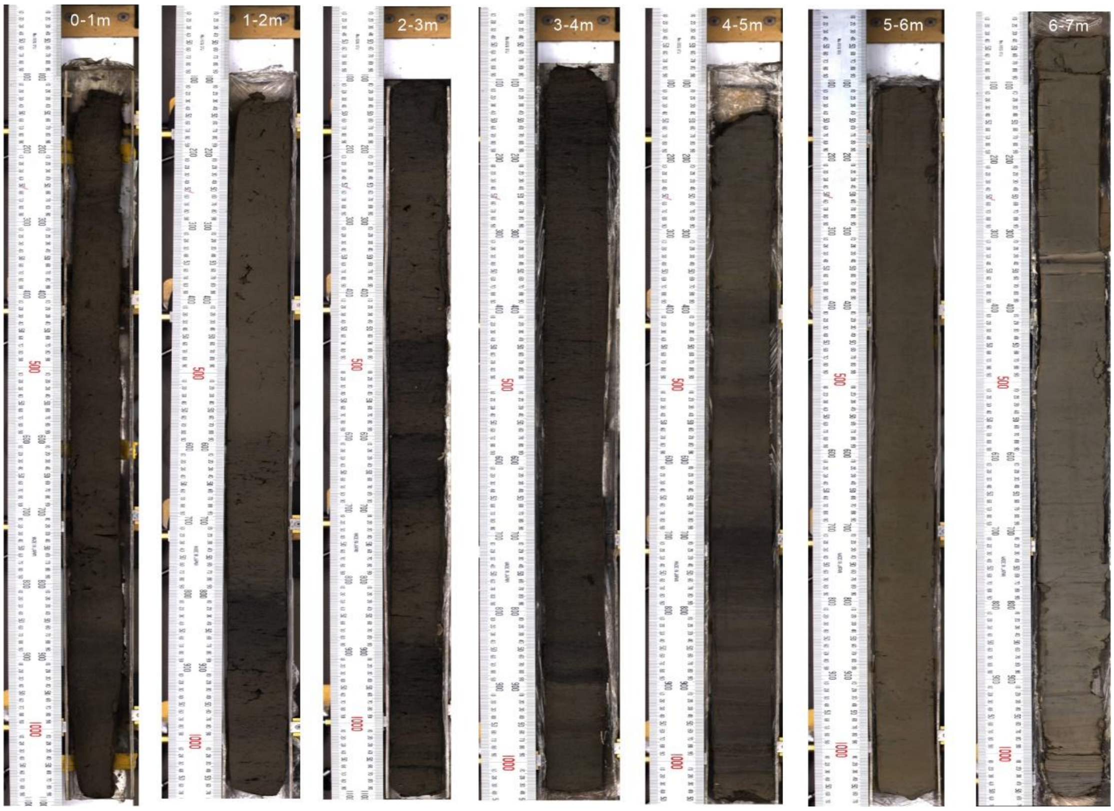

Fig. S3. Digital line-scan images of NBC21A core sections. 0-7 m; rulers with centimeter scale on left, illustrating the depositional integrity of the Nianbu Co sediment records.

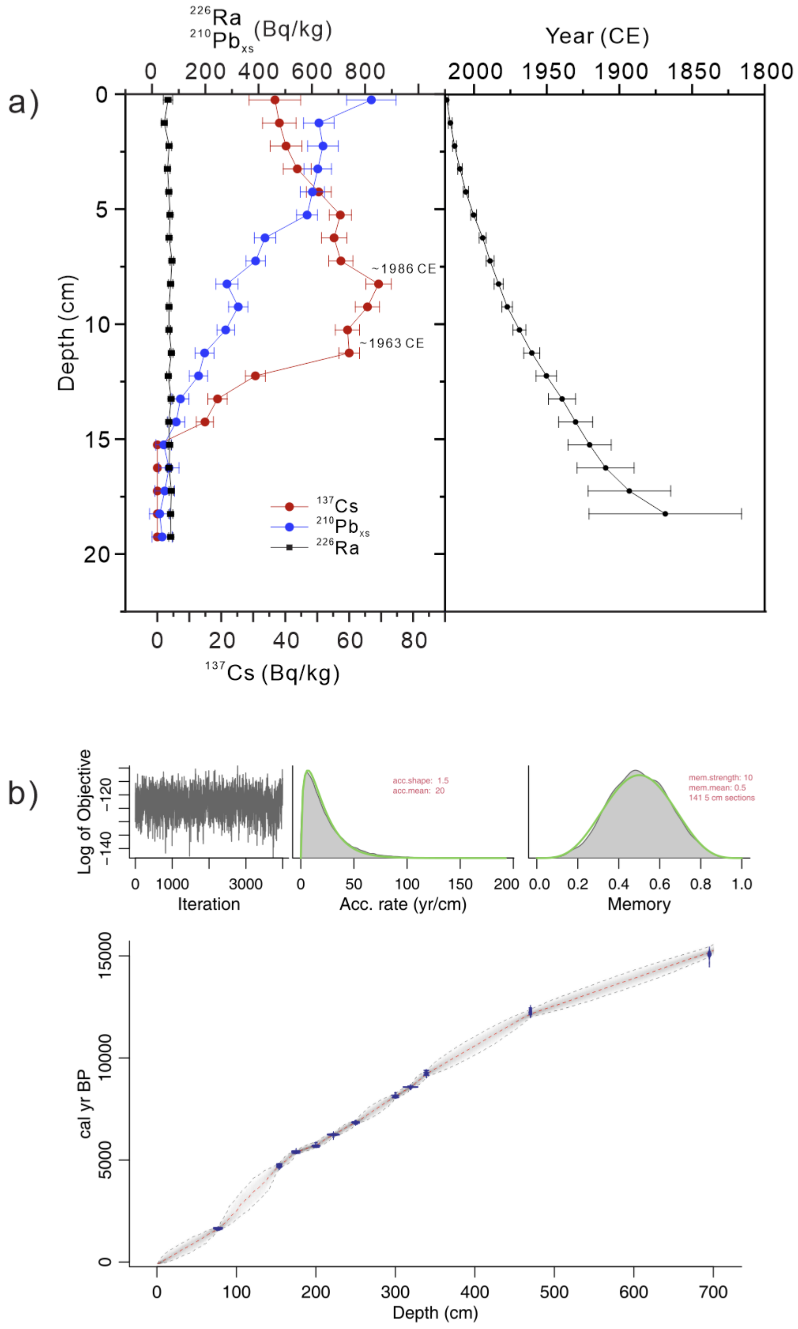

Fig. S4. Age-depth model for the surface sediment core and long cores. a) Radiometric dating analysis using gamma spectroscopy showing ^{137}Cs, ^{226}Ra and unsupported (excess) ^{210}Pb activities ($^{210}Pb_{xs}$) in becquerels per kilogram (Bq/kg) dried sediment plotted against core depth (left panel), and estimated age (CE) plotted against core depth and associated errors based on the constant rate of supply (CRS) model (right panel). **b**) Bayesian age-depth model for NBC19/21A using the ‘rbacon’ package in R. The blue areas represent the 2σ probability distributions of the calibrated ^{14}C ages, the greyscales indicate all likely age-depth models, grey dotted lines show the 95% confidence intervals, and the red dotted line shows the single "best" model based on the median age for each depth.

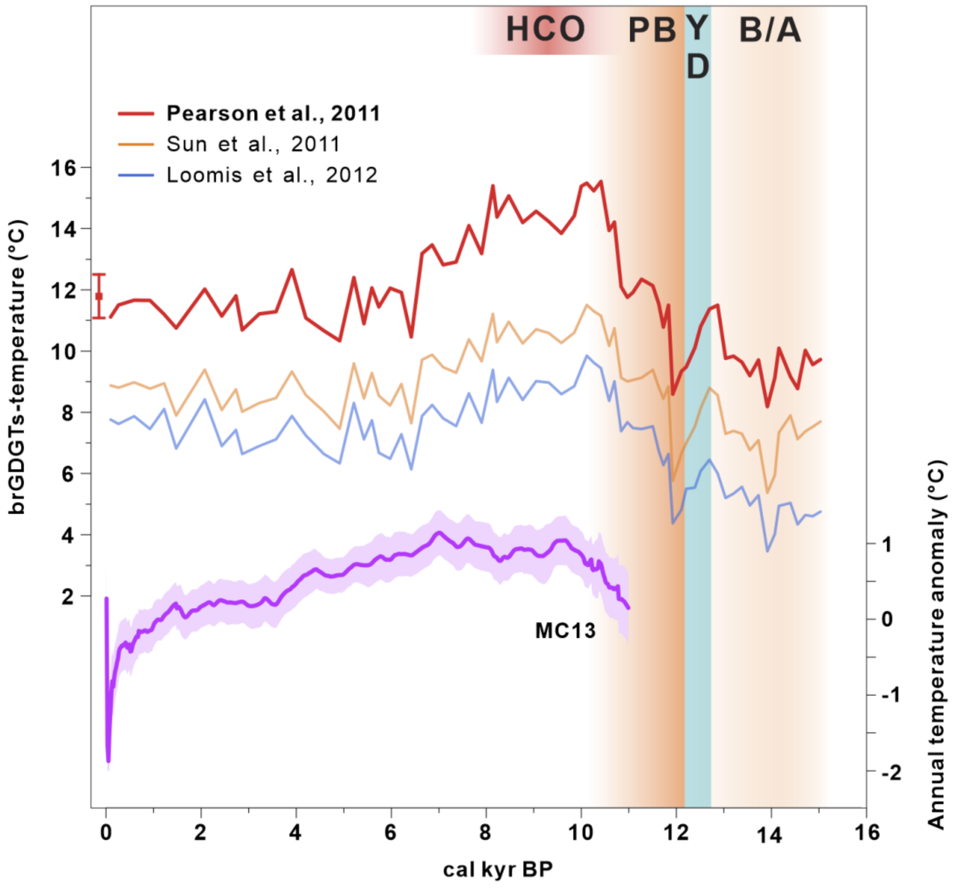

Fig. S5. Comparisons of brGDGT-based temperature variation inferred from different calibrations (*42*, *85*, *86*) **in the long sediment core from Nianbu Co.** The red error bar represents the modern instrumental mean warm-season temperature with 1 SD (April to August; 1978-2019 CE). This panel also shows a marine-dominated multiproxy stack for 30-90°N (MC13) (*43*). Vertical shading highlights the various climatic periods of this study including the warmest period (red) of the Holocene Climatic Optimum (HCO), the warm phases (orange) of the Bølling-Allerød (B/A) and the Pre-Boreal (PB), and the cold phase (blue) of the Younger Dryas (YD).

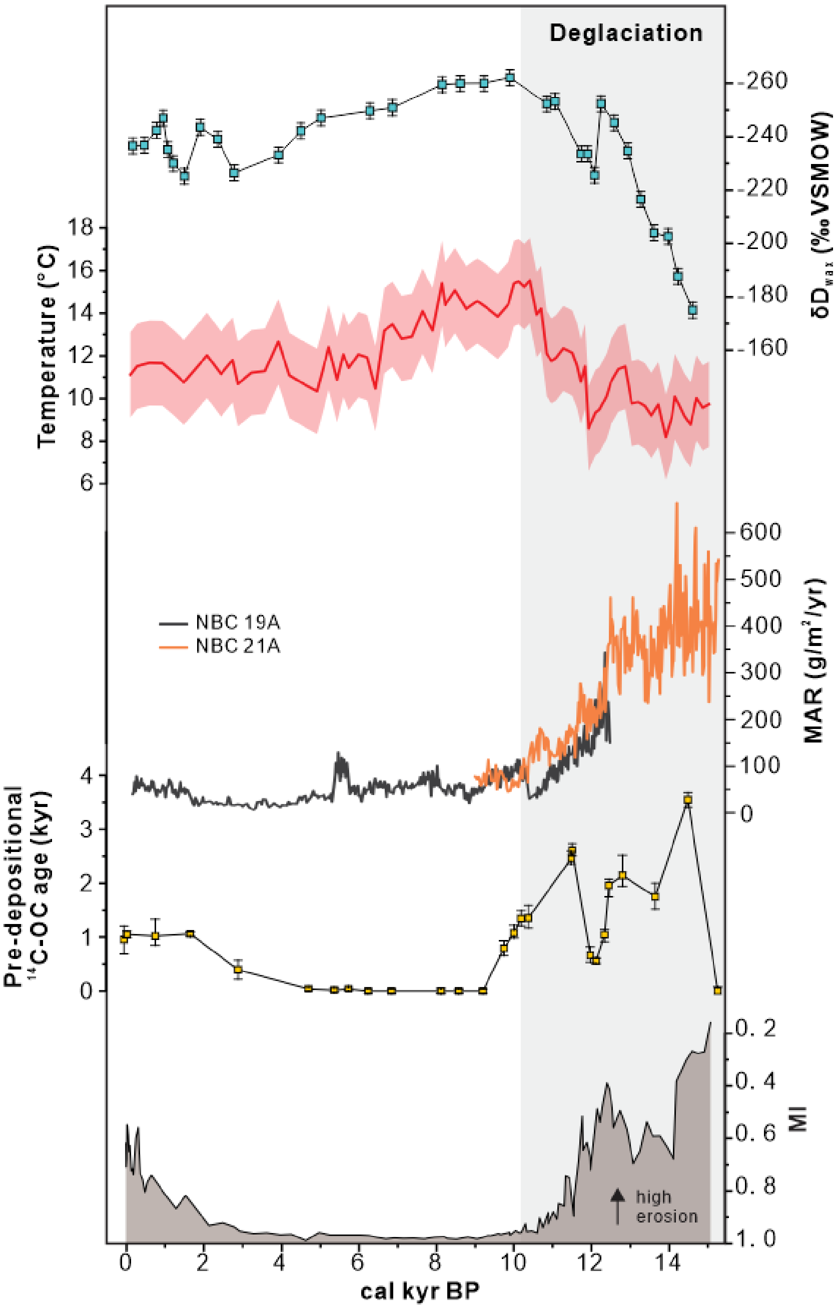

Fig. S6. Comparison climatic change with erosion-related proxies. Comparison δD_{wax} (ISM dynamics) with temperature, mass accumulation rate, and the Methane Index (MI) (*44*).

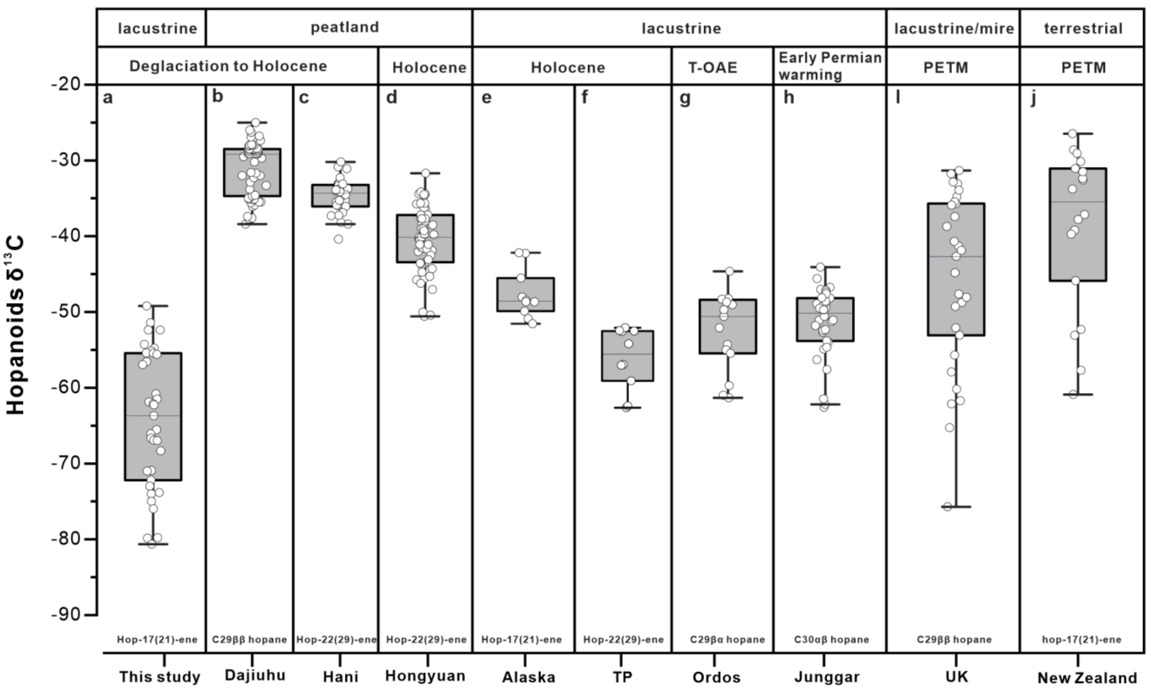

Fig. S7. Compilation of variability in hopanoid $\delta^{13}C$ values in different systems and time periods. a) This study. **b**) Dajiuhu peatland in central China (*66*). **c**) Hani peatland in northeastern China (*99*). **d**) Hongyuan peatland in eastern TP (*100*). **e**) Thermokarst lake in Alaska (*101*). **f**) Lake Koucha in eastern TP (*102*). **g**) Paleo-lake in northern China during the Toarcian Oceanic Anoxic Event (T-OAE) (*37*). **h**) Paleo-lake Junggar in northwestern China (*36*). **i**) Paleo-lacustrine/mire deposit in England (*33*). **j**) Paleo-terrestrial deposit in New Zealand (*34*).

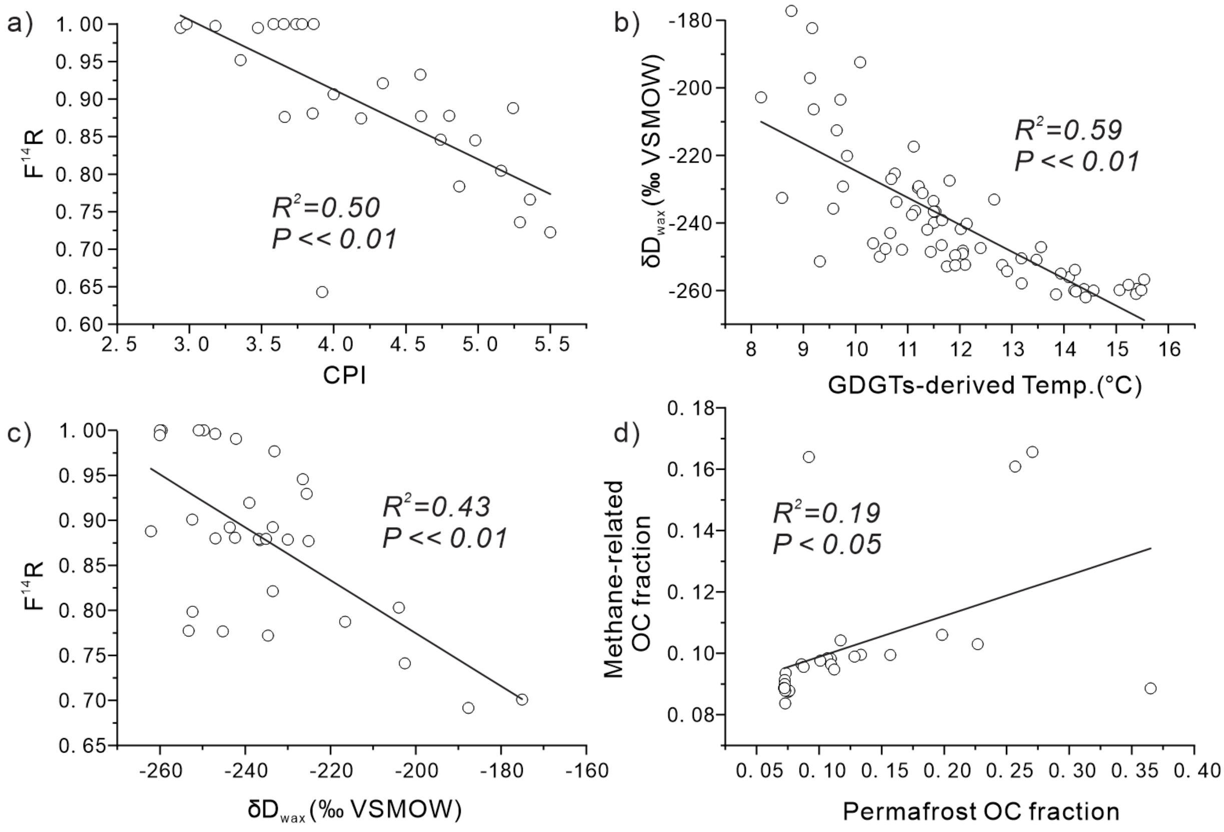

Fig. S8. Correlation between the sedimentary data. **a**) Correlation between the Carbon Preference Index (CPI) with organic-matter age structure. **b**) Cross plots of δD_{wax} against brGDGTs-derived temperatures. **c**) Correlation between δD_{wax} with organic-matter age structure. d) Cross plots of the permafrost OC fraction against the methane-related OC fraction.

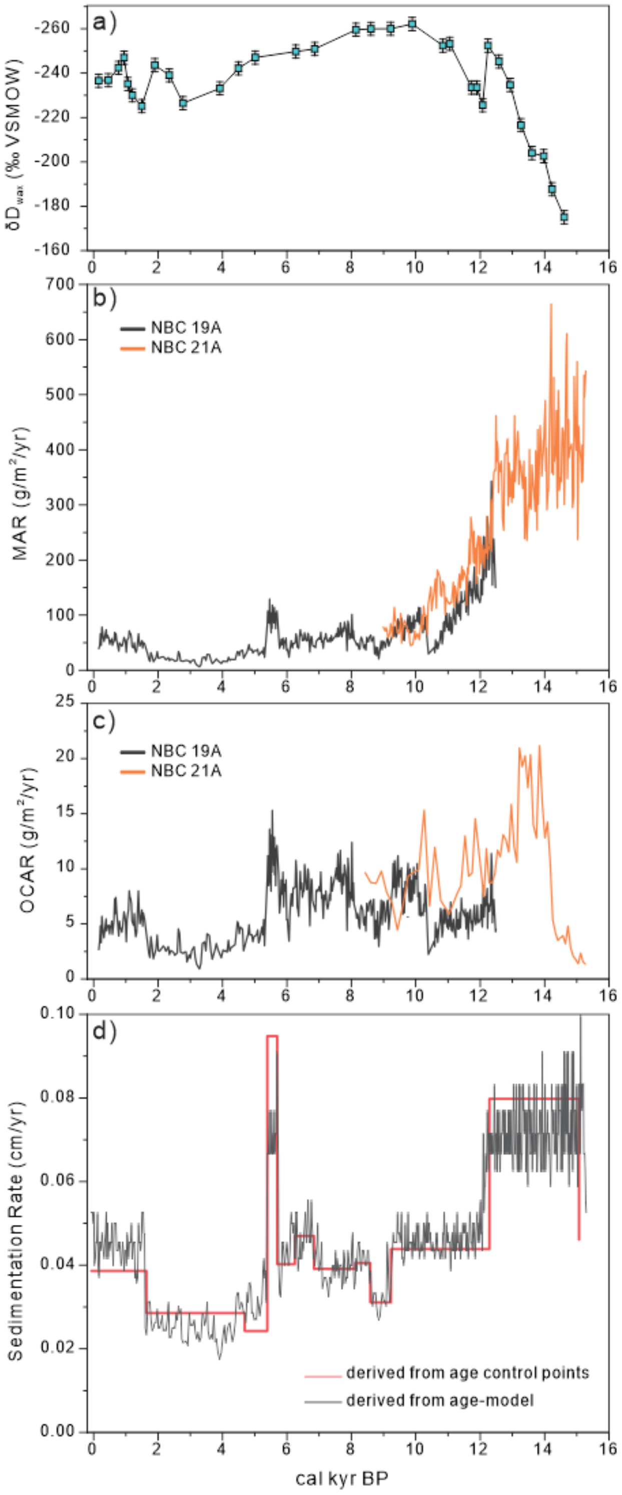

Fig. S9. Comparison of hydroclimate changes with sediment indicators. (**a**) δD$_{wax}$ trends (ISM dynamics). (**b**) mass accumulation rate (MAR). (**c**) organic carbon accumulation rate (OCAR). (**d**) Sedimentation rate.

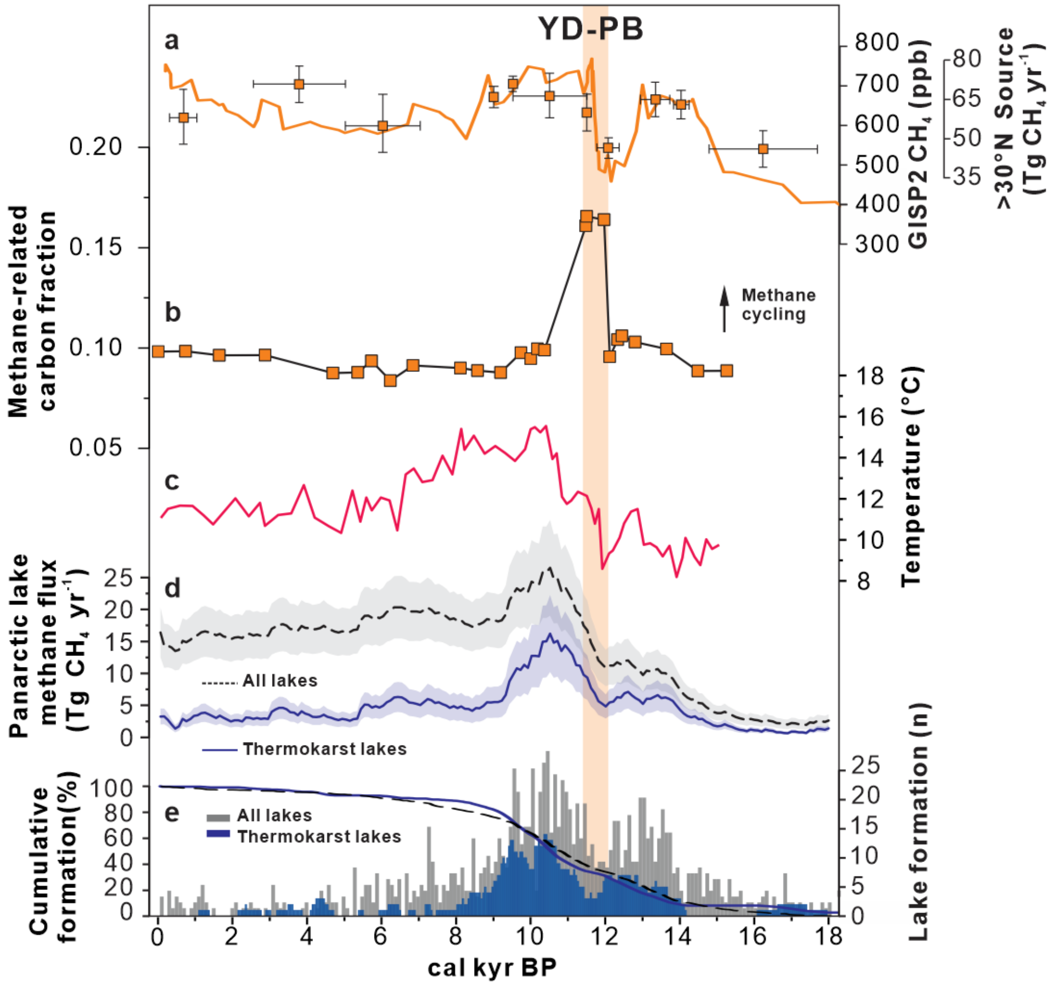

Fig. S10. Intensity of methane activity in relation to atmospheric methane concentration, temperature changes, lake formations and estimated methane emissions. (**a**), Atmospheric methane concentrations from the GISP2 ice core (*15*), also shown is the discrete northern (>30° N) source of AMC contributions (mean ± SD) inferred from the interpolar gradient (*10–12*). (**b**), Methane activity derived from the methane-related carbon fraction. (**c**), Absolute temperature variation derived from brGDGTs. (**d**), Published methane emissions estimates with temporal uncertainty and corresponding 1σ error (SD) envelopes for all pan-Arctic (grey) and thermokarst (blue) lakes (*5*) (based on rates of new thermokarst lake formation in each timestep, details see ref. (*103*). (**e**) Basal ages of thermokarst lakes used in thermokarst-lake methane emission estimates (dark blue) and of lakes used to model pan-Arctic lake methane emissions from the full suite of lake types (grey) (*5*), continuous lines in corresponding colors show cumulative formation of lakes in each dataset as a fraction of the total. Orange boxes highlight the Younger Dryas-Preboreal (YD-PB) transition.

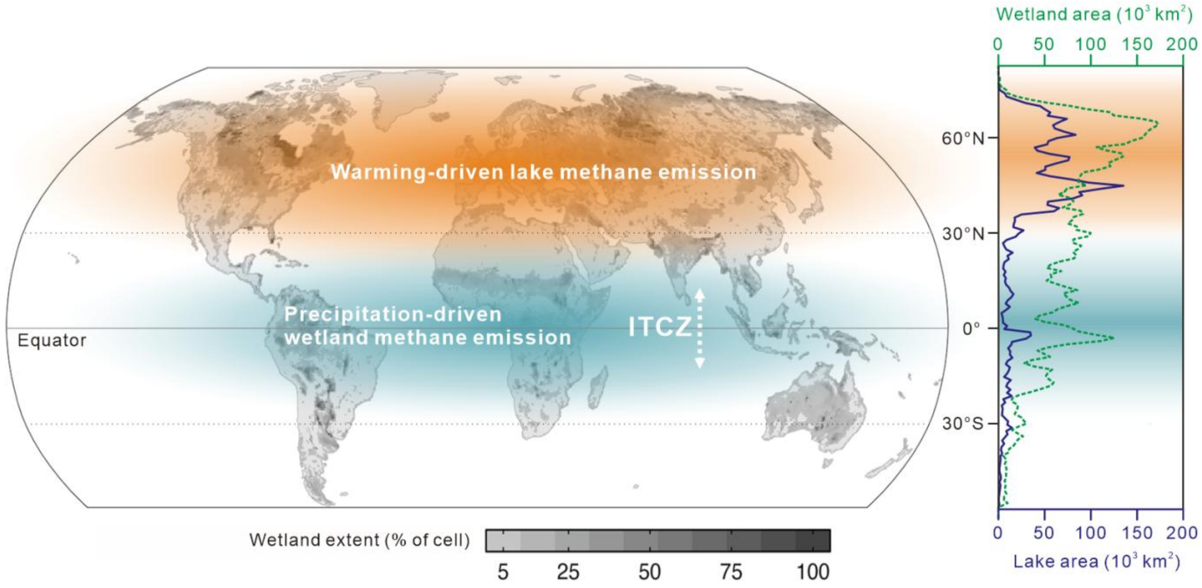

Fig. S11. Conceptual diagram depicting dual nature of deglacial methane emissions. The left panel background shows the global wetland distribution (*104*), and the conceptual diagram illustrate the warming-driven lake emission in northern extra-tropics and precipitation-driven wetland methane emission in tropics, also shown is the intertropical convergence zone (ITCZ), the tropical rainfall belt. The right panel shows zonal wetland (*105*) and lake (*106*) area distributions.

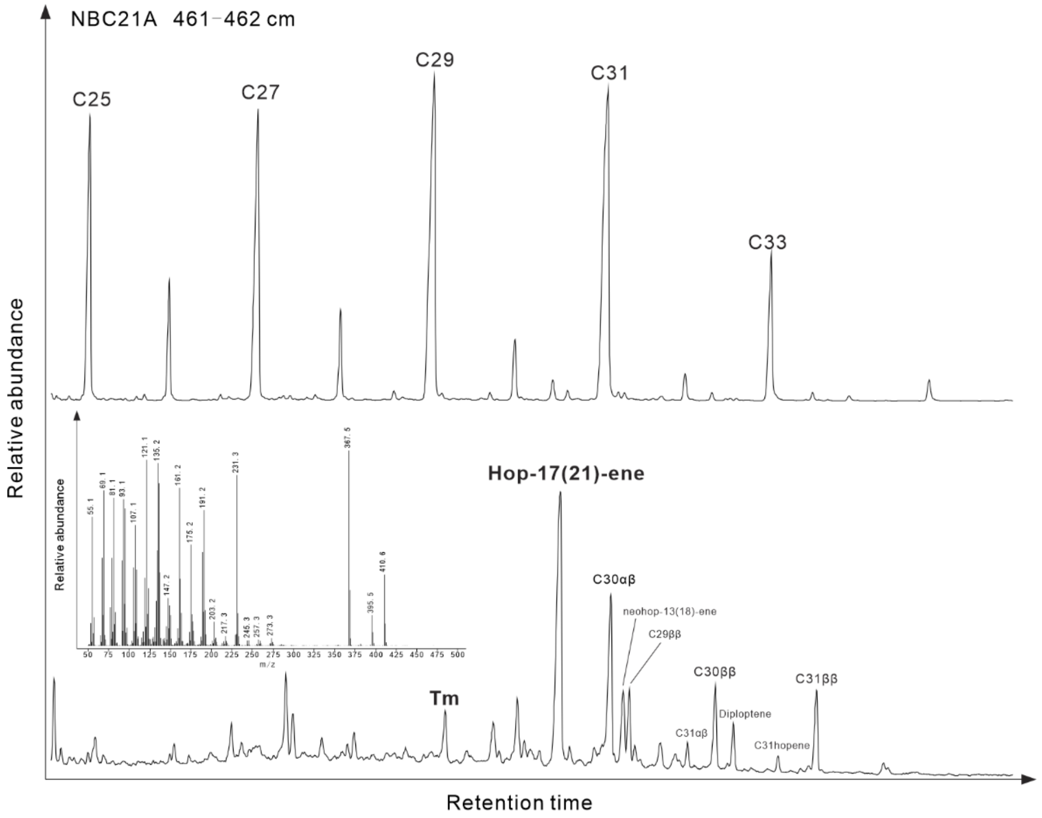

Fig. S12. Chromatograms of hydrocarbon fractions in a selected sample from NBC-19/21A cores. After treatment using the urea adduction method, the upper panel shows the total ion chromatogram (TIC) of adduct fraction, i.e., *n*-alkanes (peaks labeled with Arabic numbers refer to n-alkanes) and the lower panel shows the TIC of non-adduct fraction, i.e., mainly hopanoids compounds. Mass spectrum of hop-17(21)-ene (structure shown) obtained from gas-chromatography mass spectrometry (GC-MS) analysis is shown in the inserted figure.

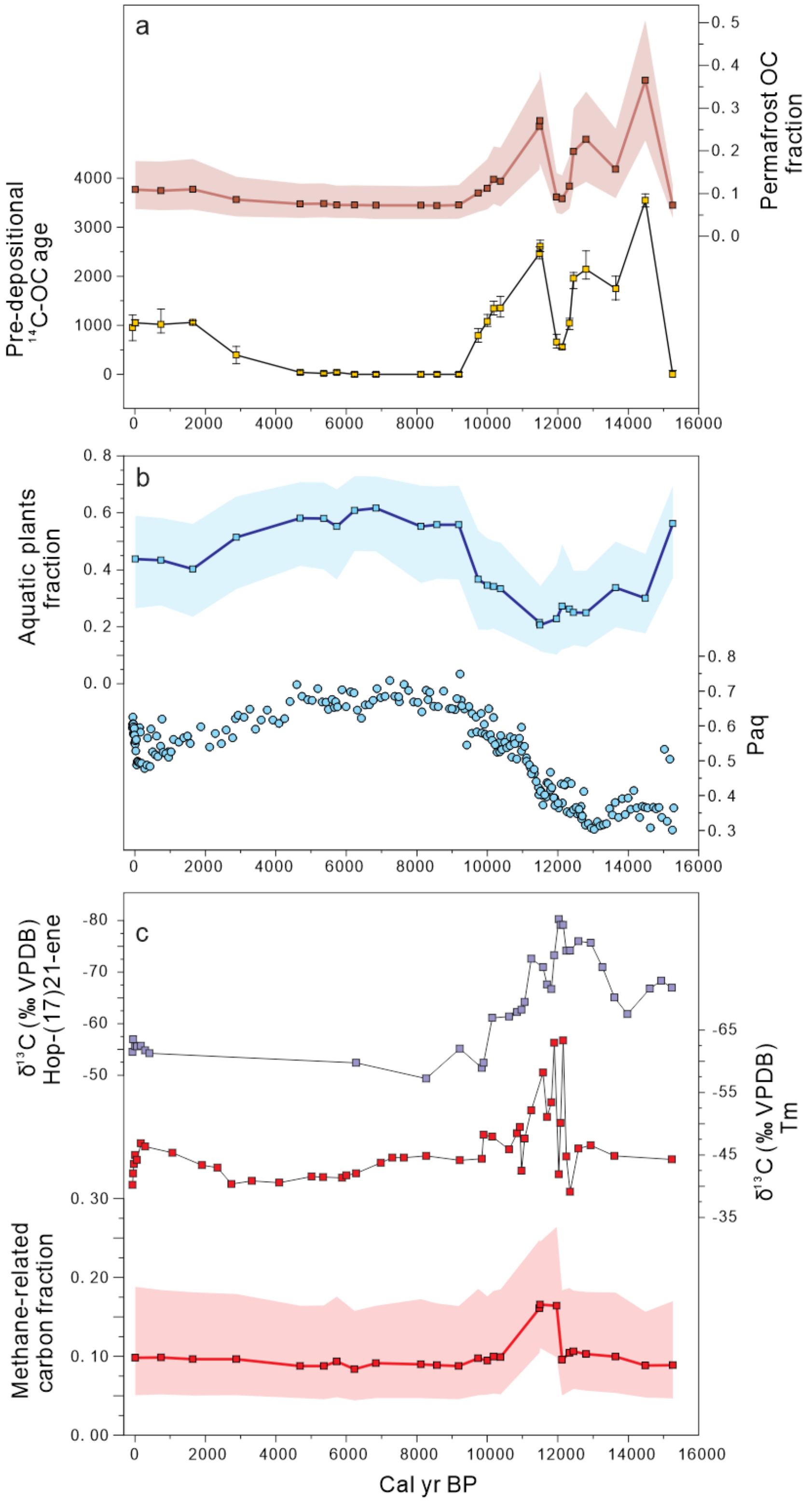

Fig. S13. Comparison of independent proxies with different OC source fraction derived from source apportionment. a) Comparison of the permafrost OC fraction with the pre-depositional ^{14}C age of OC. b) Comparison of the aquatic OC fraction with the P_{aq} index. c) Comparison of the methane-related O*C* fraction with Hop-(17)21-ene and Tm δ^{13}C values. The shading shows the interquartile range predicated by source apportionment.

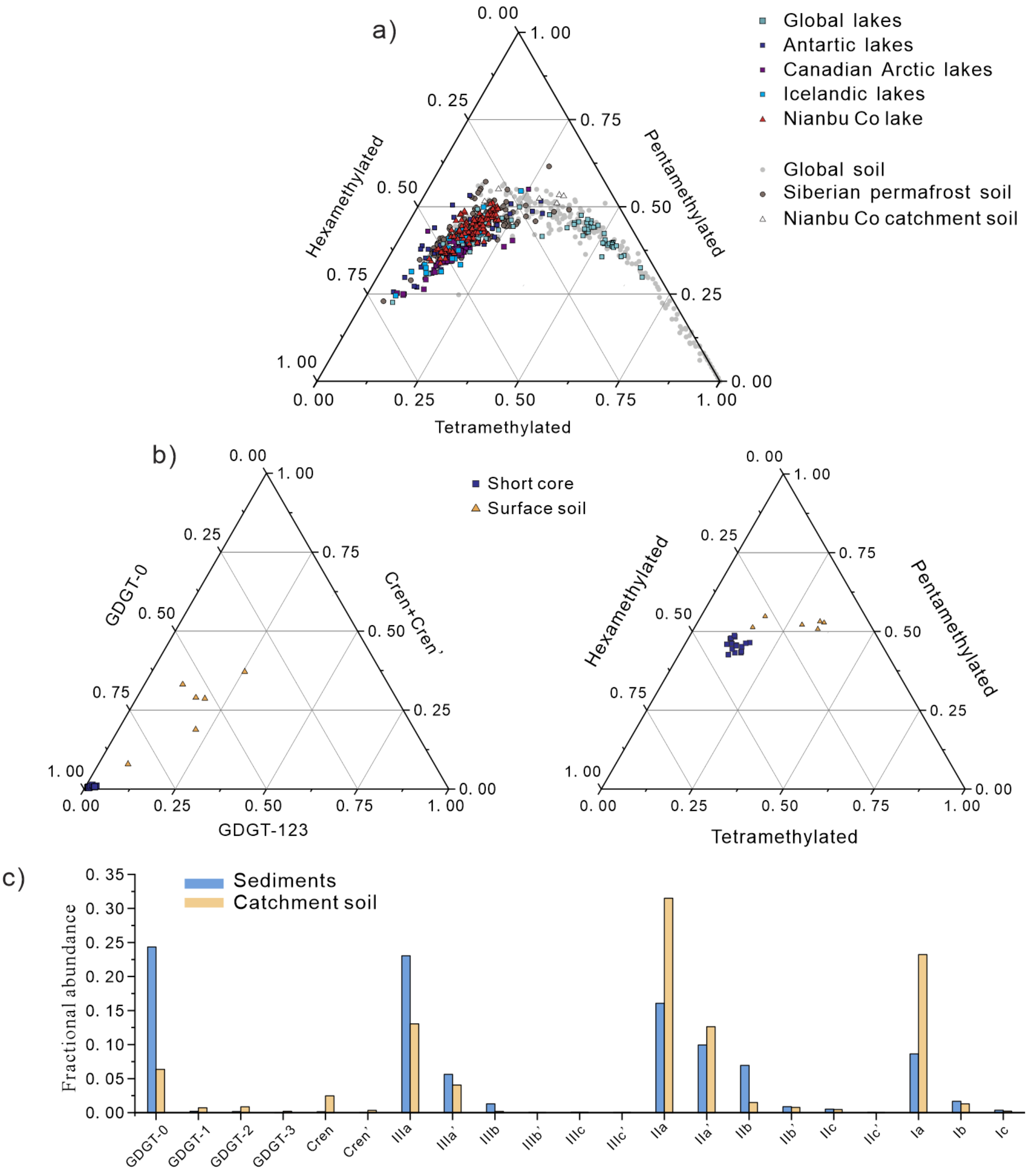

Fig. S14. GDGT composition in the Nianbu Co surface sediments and catchment soil. a) ternary diagram of the hexa-, penta- and tetra-methylated branched GDGTs in Nianbu Co sediments and in the catchment soil in comparison with global lakes (*42*), Antarctic lakes (*107*), Canadian Arctic lakes (*83*, *108*), Icelandic lakes (*108*); global soil (*109*) and Siberian permafrost soil (*110*) dataset. b) Ternary plot showing the comparison between GDGT composition (iGDGTs, left; brGDGTs, right) in surface sediments (n=16) and catchment soil (n=6). c) Fractional abundance of GDGT composition in Nianbu Co surface sediments in comparison with those in the catchment soil.

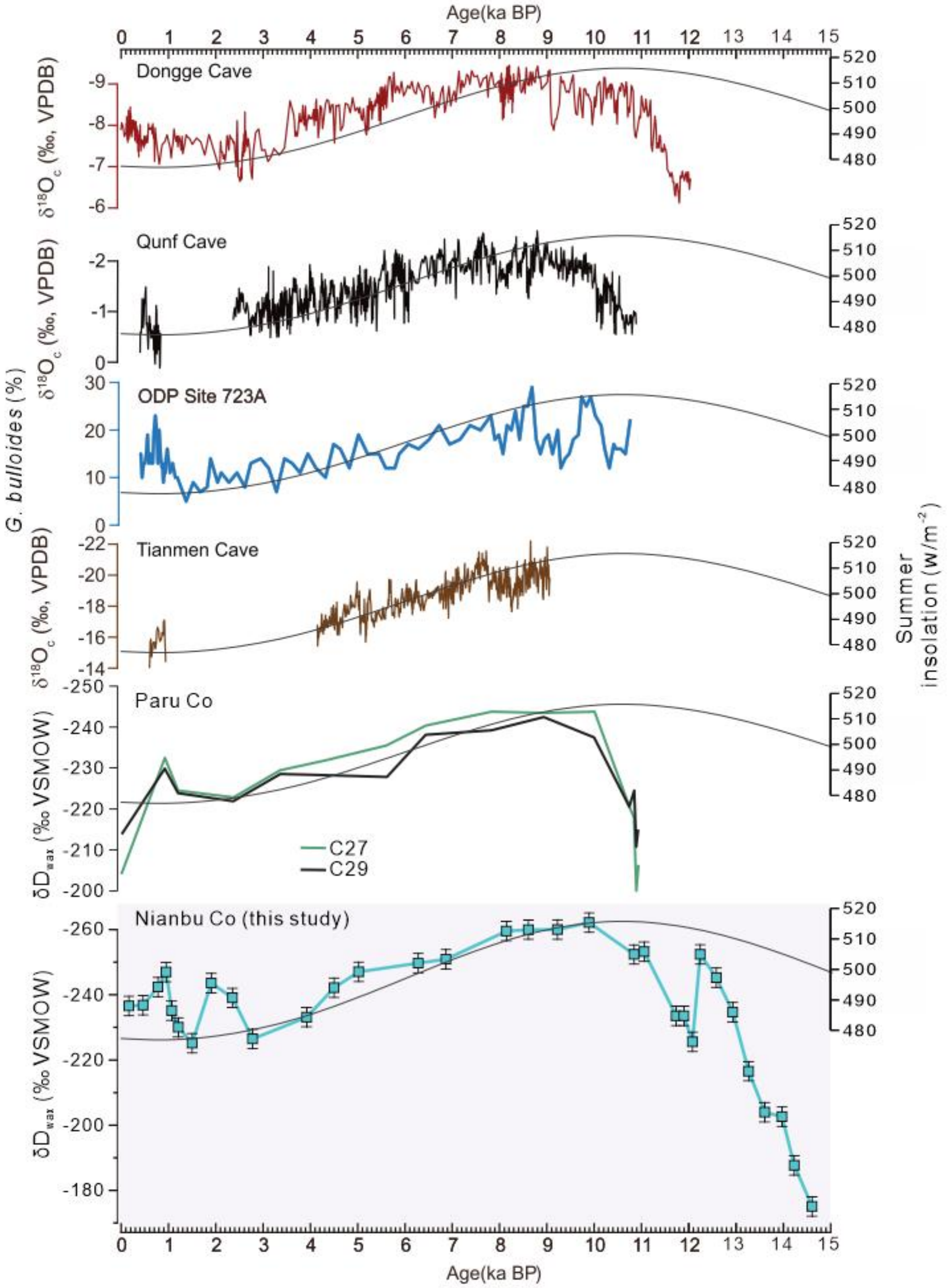

Fig. S15. Comparison of the δD_{wax} trends with other Asian summer monsoon proxy records. From top to bottom: $\delta^{18}O_c$ of Dongge Cave from southern China (*111*), $\delta^{18}O_c$ of Qunf Cave (*112*), *Globigerina bulloides* percentage in ODP Site 723A offshore Oman (*78*), $\delta^{18}O_c$ of Sahiya Cave from India (*113*), δD_{wax} of nearby lake Paru Co (*94*), δD_{wax} of Nianbu Co (this study). Also shown are the summer insolation at 30 °N (*114*).

Table S1: Age model of NBC19/21A

Sample	Lab ID	Depth (cm)	Material	^{14}C age (^{14}C years)	Age error (^{14}C years)	Calendar age lower (years BP)	Calendar age upper (years BP)	Calendar age (years BP)
NBC-1	Beta-551379	66-67	terrestrial-macrofossil	1740	30	1565	1714	1640
NBC-2	Beta-551380	153-154	terrestrial-macrofossil	4170	30	4611	4768	4690
NBC-3	Beta-551381	170-171	terrestrial-macrofossil	4660	30	5314	5468	5391
NBC-4	Beta-551382	199-200	terrestrial-macrofossil	4980	30	5641	5753	5697
NBC-5	Beta-551383	221-222	terrestrial-macrofossil	5440	40	6181	6307	6244
NBC-6	Beta-551384	249-250	terrestrial-macrofossil	6000	30	6749	6930	6840
NBC-8	Beta-551386	299-300	terrestrial-macrofossil	7340	40	8023	8213	8118
NBC-9	Beta-551387	318-319	terrestrial-macrofossil	7800	30	8536	8638	8587
NBC-10	Beta-551388	338-339	terrestrial-macrofossil	8270	30	9134	9326	9230
NBC2-9	Beta-580712	472-473	terrestrial-macrofossil	10390	30	12096	12477	12287
NBC654	Ua-78129	692-697*	BOC	12653	76	14825	15314	15070

*We assume sample 692-697cm has zero pre-depositional age and was used for age model.

Table S2: Radiocarbon data of NBC19/21A, NBC19GC2 and submerged plants

Depth (cm)	Sample	Lab ID	Material	^{14}C age (^{14}C years)	Age error (^{14}C years)
-	NBC2-1	Beta-580707	Submerged plants	1120	30
3.5-4	NBC19GC2_3.5-4cm	Beta-675071	BOC	1000	30
12.5-13	NBC19GC2_12.5-13cm	Beta-675072	BOC	1240	30
37-38	NBC19A-S1-28	LZU-20212	BOC	1870	20
76-77	NBC19A-S1-67	LZU-20213	BOC	2800	20
108-109	NBC2-3	Beta-580708	BOC	3180	30
153-154	NBC19A-S2-2-150	LZU-20214	BOC	4210	20
174-175	NBC19A-S2-2-171	LZU-20215	BOC	4680	20
199-200	NBC19A-S2-2-196	LZU-20216	BOC	5020	20
221-222	NBC19A-S3-218	LZU-20217	BOC	5230	20
249-250	NBC19A-S3-246	LZU-20218	BOC	5860	30
299-300	NBC-S3-296	LZU-20219	BOC	7340	30
318-319	NBC-S3-321	LZU-20220	BOC	7590	30
338-339	NBC-S4-341	LZU-20221	BOC	8250	30
362-363	NBC19-S4-365	LZU-20222	BOC	9560	30
374-375	NBC-11	BETA-551389	Oogonia (aquatic)	10160	30
374-375	NBC19-S4-377	LZU-20223	BOC	9970	30
382-383	NBC2-6	Beta-580709	BOC	10310	30
391-392	NBC19-S4-394	LZU-20224	BOC	10570	40
441-442	NBC2-7	Beta - 580710	BOC	12490	40
442-443	NBC19-S5-437	LZU-20225	BOC	12640	40
464-465	NBC19-S5-460	LZU-20226	BOC	10940	30

472-473	NBC2-8	Beta-580711	BOC	10950	40
486-487	NBC19-S5-482	LZU-20227	BOC	11430	30
494-495	NBC19-S5-490	LZU-20228	BOC	12360	40
519-520	NBC507	Ua-77596	BOC	13056	48
579-580	NBC557	Ua-78127	BOC	13560	46
639-640	NBC607	Ua-78128	BOC	15966	52

REFERENCES AND NOTES

1. N. L. Bindoff, P. A. Stott, K. M. AchutaRao, M. R. Allen, N. Gillett, D. Gutzler, K. Hansingo, G. Hegerl, Y. Hu, S. Jain, I. I. Mokhov, J. Overland, J. Perlwitz, R. Sebbari, X. Zhang, "Detection and attribution of climate change: From global to regional" in *Climate Change 2013: The Physical Science Basis. Contribution of Working Group I to the Fifth Assessment Report of the Intergovernmental Panel on Climate Change*, T. F. Stocker, D. Qin, G.-K. Plattner, M. Tignor, S. K. Allen, J. Boschung, A. Nauels, Y. Xia, V. Bex, P. M. Midgley, Eds. (Cambridge Univ. Press, 2013), pp. 867–952.

2. M. N. Dyonisius, V. V. Petrenko, A. M. Smith, Q. Hua, B. Yang, J. Schmitt, J. Beck, B. Seth, M. Bock, B. Hmiel, I. Vimont, J. A. Menking, S. A. Shackleton, D. Baggenstos, T. K. Bauska, R. H. Rhodes, P. Sperlich, R. Beaudette, C. Harth, M. Kalk, E. J. Brook, H. Fischer, J. P. Severinghaus, R. F. Weiss, Old carbon reservoirs were not important in the deglacial methane budget. *Science* **367**, 907–910 (2020).

3. V. V. Petrenko, A. M. Smith, H. Schaefer, K. Riedel, E. Brook, D. Baggenstos, C. Harth, Q. Hua, C. Buizert, A. Schilt, X. Fain, L. Mitchell, T. Bauska, A. Orsi, R. F. Weiss, J. P. Severinghaus, Minimal geological methane emissions during the Younger Dryas–Preboreal abrupt warming event. *Nature* **548**, 443–446 (2017).

4. B. Hmiel, V. V. Petrenko, M. N. Dyonisius, C. Buizert, A. M. Smith, P. F. Place, C. Harth, R. Beaudette, Q. Hua, B. Yang, I. Vimont, S. E. Michel, J. P. Severinghaus, D. Etheridge, T. Bromley, J. Schmitt, X. Faïn, R. F. Weiss, E. Dlugokencky, Preindustrial $^{14}CH_4$ indicates greater anthropogenic fossil CH_4 emissions. *Nature* **578**, 409–412 (2020).

5. L. S. Brosius, K. M. Walter Anthony, C. C. Treat, M. C. Jones, M. Dyonisius, G. Grosse, Panarctic lakes exerted a small positive feedback on early Holocene warming due to deglacial release of methane. *Commun. Earth Environ.* **4**, 271 (2023).

6. A. Quiquet, A. T. Archibald, A. D. Friend, J. Chappellaz, J. G. Levine, E. J. Stone, P. J. Telford, J. A. Pyle, The relative importance of methane sources and sinks over the Last Interglacial period and into the last glaciation. *Quat. Sci. Rev.* **112**, 1–16 (2015).

7. B. Riddell-Young, J. E. Lee, E. J. Brook, J. Schmitt, H. Fischer, T. K. Bauska, J. A. Menking, R. Iseli, J. R. Clark, Abrupt changes in biomass burning during the last glacial period. *Nature* **637**, 91–96 (2025).

8. J. S. Singarayer, P. J. Valdes, P. Friedlingstein, S. Nelson, D. J. Beerling, Late Holocene methane rise caused by orbitally controlled increase in tropical sources. *Nature* **470**, 82–85 (2011).

9. B. Riddell-Young, J. Rosen, E. Brook, C. Buizert, K. Martin, J. Lee, J. Edwards, M. Mühl, J. Schmitt, H. Fischer, T. Blunier, Atmospheric methane variability through the Last Glacial Maximum and deglaciation mainly controlled by tropical sources. *Nat. Geosci.* **16**, 1174–1180 (2023).

10. M. Baumgartner, A. Schilt, O. Eicher, J. Schmitt, J. Schwander, R. Spahni, H. Fischer, T. F. Stocker, High-resolution interpolar difference of atmospheric methane around the Last Glacial Maximum. *Biogeosciences* **9**, 3961–3977 (2012).

11. J. Chappellaz, T. Blunier, S. Kints, A. Dällenbach, J.-M. Barnola, J. Schwander, D. Raynaud, B. Stauffer, Changes in the atmospheric CH_4 gradient between Greenland and Antarctica during the Holocene. *J. Geophys. Res. Atmos.* **102**, 15987–15997 (1997).

12. J.-W. Yang, J. Ahn, E. J. Brook, Y. Ryu, Atmospheric methane control mechanisms during the early Holocene. *Clim. Past* **13**, 1227–1242 (2017).

13. A. Dällenbach, T. Blunier, J. Flückiger, B. Stauffer, J. Chappellaz, D. Raynaud, Changes in the atmospheric CH_4 gradient between Greenland and Antarctica during the Last Glacial and the transition to the Holocene. *Geophys. Res. Lett.* **27**, 1005–1008 (2000).

14. A. V. Reyes, C. A. Cooke, Northern peatland initiation lagged abrupt increases in deglacial atmospheric CH_4. *Proc. Natl. Acad. Sci. U.S.A.* **108**, 4748–4753 (2011).

15. E. J. Brook, S. Harder, J. Severinghaus, E. J. Steig, C. M. Sucher, On the origin and timing of rapid changes in atmospheric methane during the Last Glacial Period. *Global Biogeochem. Cycles* **14**, 559–572 (2000).

16. T. Sowers, Late quaternary atmospheric CH_4 isotope record suggests marine clathrates are stable. *Science* **311**, 838–840 (2006).

17. J. R. Melton, H. Schaefer, M. J. Whiticar, Enrichment in ^{13}C of atmospheric CH_4 during the Younger Dryas termination. *Clim. Past* **8**, 1177–1197 (2012).

18. C. C. Treat, M. C. Jones, L. Brosius, G. Grosse, K. Walter Anthony, S. Frolking, The role of wetland expansion and successional processes in methane emissions from northern wetlands during the Holocene. *Quat. Sci. Rev.* **257**, 106864 (2021).

19. Y. Zhu, K. J. Purdy, Ö. Eyice, L. Shen, S. F. Harpenslager, G. Yvon-Durocher, A. J. Dumbrell, M. Trimmer, Disproportionate increase in freshwater methane emissions induced by experimental warming. *Nat. Clim. Chang.* **10**, 685–690 (2020).

20. G. Yvon-Durocher, A. P. Allen, D. Bastviken, R. Conrad, C. Gudasz, A. St-Pierre, N. Thanh-Duc, P. A. del Giorgio, Methane fluxes show consistent temperature dependence across microbial to ecosystem scales. *Nature* **507**, 488–491 (2014).

21. R. C. H. Aben, N. Barros, E. van Donk, T. Frenken, S. Hilt, G. Kazanjian, L. P. M. Lamers, E. T. H. M. Peeters, J. G. M. Roelofs, L. N. de Senerpont Domis, S. Stephan, M. Velthuis, D. B. Van de Waal, M. Wik, B. F. Thornton, J. Wilkinson, T. DelSontro, S. Kosten, Cross continental increase in methane ebullition under climate change. *Nat. Commun.* **8**, 1682 (2017).

22. J. B. Emerson, R. K. Varner, M. Wik, D. H. Parks, R. B. Neumann, J. E. Johnson, C. M. Singleton, B. J. Woodcroft, R. Tollerson, A. Owusu-Dommey, M. Binder, N. L. Freitas, P. M. Crill, S. R. Saleska, G. W. Tyson, V. I. Rich, Diverse sediment microbiota shape methane emission temperature sensitivity in Arctic lakes. *Nat. Commun.* **12**, 5815 (2021).

23. J. Obu, S. Westermann, A. Bartsch, N. Berdnikov, H. H. Christiansen, A. Dashtseren, R. Delaloye, B. Elberling, B. Etzelmüller, A. Kholodov, A. Khomutov, A. Kääb, M. O. Leibman, A. G. Lewkowicz, S. K. Panda, V. Romanovsky, R. G. Way, A. Westergaard-Nielsen, T. Wu, J. Yamkhin, D. Zou, Northern Hemisphere permafrost map based on TTOP modelling for 2000–2016 at 1 km^2 scale. *Earth-Sci. Rev.* **193**, 299–316 (2019).

24. D. Olefeldt, S. Goswami, G. Grosse, D. Hayes, G. Hugelius, P. Kuhry, A. D. McGuire, V. E. Romanovsky, A. B. K. Sannel, E. A. G. Schuur, M. R. Turetsky, Circumpolar distribution and carbon storage of thermokarst landscapes. *Nat. Commun.* **7**, 13043 (2016).

25. K. Walter Anthony, R. Daanen, P. Anthony, T. Schneider von Deimling, C.-L. Ping, J. P. Chanton, G. Grosse, Methane emissions proportional to permafrost carbon thawed in Arctic lakes since the 1950s. *Nat. Geosci.* **9**, 679–682 (2016).

26. G. Yang, Z. Zheng, B. Abbott, D. Olefeldt, C. Knoblauch, Y. Song, L. Kang, S. Qin, Y. Peng, Y. Yang, Characteristics of methane emissions from alpine thermokarst lakes on the Tibetan Plateau. *Nat. Commun.* **14**, 3121 (2023).

27. K. M. Walter, S. A. Zimov, J. P. Chanton, D. Verbyla, F. S. Chapin III, Methane bubbling from Siberian thaw lakes as a positive feedback to climate warming. *Nature* **443**, 71–75 (2006).

28. T. W. Drake, K. P. Wickland, R. G. M. Spencer, D. M. McKnight, R. G. Striegl, Ancient low–molecular-weight organic acids in permafrost fuel rapid carbon dioxide production upon thaw. *Proc. Natl. Acad. Sci. U.S.A.* **112**, 13946–13951 (2015).

29. J. Martens, B. Wild, F. Muschitiello, M. O'Regan, M. Jakobsson, I. Semiletov, O. V. Dudarev, Ö. Gustafsson, Remobilization of dormant carbon from Siberian-Arctic permafrost during three past warming events. *Sci. Adv.* **6**, eabb6546 (2020).

30. M. Winterfeld, G. Mollenhauer, W. Dummann, P. Köhler, L. Lembke-Jene, V. D. Meyer, J. Hefter, C. McIntyre, L. Wacker, U. Kokfelt, R. Tiedemann, Deglacial mobilization of pre-aged terrestrial carbon from degrading permafrost. *Nat. Commun.* **9**, 3666 (2018).

31. J. Wu, G. Mollenhauer, R. Stein, P. Köhler, J. Hefter, K. Fahl, H. Grotheer, B. Wei, S.-I. Nam, Deglacial release of petrogenic and permafrost carbon from the Canadian Arctic impacting the carbon cycle. *Nat. Commun.* **13**, 7172 (2022).

32. K.-U. Hinrichs, L. R. Hmelo, S. P. Sylva, Molecular fossil record of elevated methane levels in late pleistocene coastal waters. *Science* **299**, 1214–1217 (2003).

33. R. D. Pancost, D. S. Steart, L. Handley, M. E. Collinson, J. J. Hooker, A. C. Scott, N. V. Grassineau, I. J. Glasspool, Increased terrestrial methane cycling at the Palaeocene–Eocene thermal maximum. *Nature* **449**, 332–335 (2007).

34. G. N. Inglis, M. Rohrssen, E. M. Kennedy, E. M. Crouch, J. I. Raine, D. P. Strogen, B. D. A. Naafs, M. E. Collinson, R. D. Pancost, Terrestrial methane cycle perturbations during the onset of the Paleocene-Eocene Thermal Maximum. *Geology* **49**, 520–524 (2021).

35. B. Kim, Y. G. Zhang, Methane hydrate dissociation across the Oligocene–Miocene boundary. *Nat. Geosci.* **15**, 203–209 (2022).

36. F. Sun, W. Hu, J. Cao, X. Wang, Z. Zhang, J. Ramezani, S. Shen, Sustained and intensified lacustrine methane cycling during Early Permian climate warming. *Nat. Commun.* **13**, 4856 (2022).

37. Y. Huang, X. Jin, R. D. Pancost, D. B. Kemp, B. D. A. Naafs, An intensified lacustrine methane cycle during the Toarcian OAE (Jenkyns Event) in the Ordos Basin, northern China. *Earth Planet. Sci. Lett.* **639**, 118766 (2024).

38. J. F. van Winden, H. M. Talbot, G.-J. Reichart, N. P. McNamara, A. Benthien, J. S. Sinninghe Damsté, Influence of temperature on the $\delta^{13}C$ values and distribution of methanotroph-related hopanoids in *Sphagnum*-dominated peat bogs. *Geobiology* **18**, 497–507 (2020).

39. N. T. Smit, D. Rush, D. X. Sahonero-Canavesi, M. Verweij, O. Rasigraf, S. Guerrero Cruz, M. S. M. Jetten, J. S. Sinninghe Damsté, S. Schouten, Demethylated hopanoids in 'Ca. Methylomirabilis oxyfera' as biomarkers for environmental nitrite-dependent methane oxidation. *Org. Geochem.* **137**, 103899 (2019).

40. F. Sun, G. Luo, R. D. Pancost, Z. Dong, Z. Li, H. Wang, Z.-Q. Chen, S. Xie, Methane fueled lake pelagic food webs in a Cretaceous greenhouse world. *Proc. Natl. Acad. Sci. U.S.A.* **121**, e2411413121 (2024).

41. Y. Wei, X. Yang, X. Qiu, H. Wei, C. Tang, Spatio-temporal variations of atmospheric methane and its response to climate on the Tibetan Plateau from 2010 to 2022. *Atmos. Environ.* **314**, 120088 (2023).

42. E. J. Pearson, S. Juggins, H. M. Talbot, J. Weckström, P. Rosén, D. B. Ryves, S. J. Roberts, R. Schmidt, A lacustrine GDGT-temperature calibration from the Scandinavian Arctic to Antarctic: Renewed potential for the application of GDGT-paleothermometry in lakes. *Geochim. Cosmochim. Acta* **75**, 6225–6238 (2011).

43. S. A. Marcott, J. D. Shakun, P. U. Clark, A. C. Mix, A reconstruction of regional and global temperature for the past 11,300 years. *Science* **339**, 1198–1201 (2013).

44. Y. G. Zhang, C. L. Zhang, X.-L. Liu, L. Li, K.-U. Hinrichs, J. E. Noakes, Methane Index: A tetraether archaeal lipid biomarker indicator for detecting the instability of marine gas hydrates. *Earth Planet. Sci. Lett.* **307**, 525–534 (2011).

45. M. P. Cooke, H. M. Talbot, P. Farrimond, Bacterial populations recorded in bacteriohopanepolyol distributions in soils from Northern England. *Org. Geochem.* **39**, 1347–1358 (2008).

46. Y. Weber, J. S. Sinninghe Damsté, J. Zopfi, C. De Jonge, A. Gilli, C. J. Schubert, F. Lepori, M. F. Lehmann, H. Niemann, Redox-dependent niche differentiation provides evidence for multiple bacterial sources of glycerol tetraether lipids in lakes. *Proc. Natl. Acad. Sci. U.S.A.* **115**, 10926–10931 (2018).

47. F. A. McInerney, S. L. Wing, The Paleocene-Eocene Thermal Maximum: A perturbation of carbon cycle, climate, and biosphere with implications for the future. *Annu. Rev. Earth Planet. Sci.* **39**, 489–516 (2011).

48. R. M. DeConto, S. Galeotti, M. Pagani, D. Tracy, K. Schaefer, T. Zhang, D. Pollard, D. J. Beerling, Past extreme warming events linked to massive carbon release from thawing permafrost. *Nature* **484**, 87–91 (2012).

49. W. E. West, K. P. Creamer, S. E. Jones, Productivity and depth regulate lake contributions to atmospheric methane. *Limnol. Oceanogr.* **61**, S51–S61 (2016).

50. K. J. Ficken, B. Li, D. L. Swain, G. Eglinton, An n-alkane proxy for the sedimentary input of submerged/floating freshwater aquatic macrophytes. *Org. Geochem.* **31**, 745–749 (2000).

51. D. Kaufman, N. McKay, C. Routson, M. Erb, C. Dätwyler, P. S. Sommer, O. Heiri, B. Davis, Holocene global mean surface temperature, a multi-method reconstruction approach. *Sci. Data* **7**, 201 (2020).

52. C. Mu, M. Mu, X. Wu, L. Jia, C. Fan, X. Peng, C. Ping, Q. Wu, C. Xiao, J. Liu, High carbon emissions from thermokarst lakes and their determinants in the Tibet Plateau. *Glob. Chang. Biol.* **29**, 2732–2745 (2023).

53. C. D. Elder, X. Xu, J. Walker, J. L. Schnell, K. M. Hinkel, A. Townsend-Small, C. D. Arp, J. W. Pohlman, B. V. Gaglioti, C. I. Czimczik, Greenhouse gas emissions from diverse Arctic Alaskan lakes are dominated by young carbon. *Nat. Clim. Chang.* **8**, 166–171 (2018).

54. K. Yuan, F. Li, G. McNicol, M. Chen, A. Hoyt, S. Knox, W. J. Riley, R. Jackson, Q. Zhu, Boreal–Arctic wetland methane emissions modulated by warming and vegetation activity. *Nat. Clim. Chang.* **14**, 282–288 (2024).

55. B. F. Thornton, G. Etiope, S. Schwietzke, A. V. Milkov, R. W. Klusman, A. Judd, D. Z. Oehler, Conflicting estimates of natural geologic methane emissions. *Elementa (Wash D C)* **9**, 00031 (2021).

56. G. Etiope, S. Schwietzke, Global geological methane emissions: An update of top-down and bottom-up estimates. *Elementa (Wash D C)* **7**, 47 (2019).

57. B. S. Lecavalier, D. A. Fisher, G. A. Milne, B. M. Vinther, L. Tarasov, P. Huybrechts, D. Lacelle, B. Main, J. Zheng, J. Bourgeois, A. S. Dyke, High Arctic Holocene temperature record from the Agassiz ice cap and Greenland ice sheet evolution. *Proc. Natl. Acad. Sci. U.S.A.* **114**, 5952–5957 (2017).

58. C. Shen, "Millennial-scale variations and centennial scale events in the Southwest Asian monsoon: Pollen evidence from Tibet,", thesis, Louisiana State University (2003).

59. P. G. Appleby, "Chronostratigraphic techniques in recent sediments" in *Tracking Environmental Change Using Lake Sediments* (Springer, 2002), pp. 171–203.

60. C. B. Ramsey, Bayesian analysis of radiocarbon dates. *Radiocarbon* **51**, 337–360 (2009).

61. P. J. Reimer, W. E. N. Austin, E. Bard, A. Bayliss, P. G. Blackwell, C. B. Ramsey, M. Butzin, H. Cheng, R. L. Edwards, M. Friedrich, P. M. Grootes, T. P. Guilderson, I. Hajdas, T. J. Heaton, A. G. Hogg, K. A. Hughen, B. Kromer, S. W. Manning, R. Muscheler, J. G. Palmer, C. Pearson, J. van der Plicht, R. W. Reimer, D. A. Richards, E. M. Scott, J. R. Southon, C. S. M. Turney, L. Wacker, F. Adolphi, U. Büntgen, M. Capano, S. M. Fahrni, A. Fogtmann-Schulz, R. Friedrich, P. Köhler, S. Kudsk, F. Miyake, J. Olsen, F. Reinig, M. Sakamoto, A. Sookdeo, S. Talamo, The IntCal20 Northern Hemisphere Radiocarbon age calibration curve (0–55 cal kBP). *Radiocarbon* **62**, 725–757 (2020).

62. M. Blaauw, J. A. Christen, Flexible paleoclimate age-depth models using an autoregressive gamma process. *Bayesian Anal.* **6**, 457–474 (2011).

63. Y. J. Wang, H. Cheng, R. L. Edwards, Z. S. An, J. Y. Wu, C.-C. Shen, J. A. Dorale, A high-resolution absolute-dated late pleistocene monsoon record from Hulu Cave, China. *Science* **294**, 2345–2348 (2001).

64. Y. He, J. Hou, M. Wang, X. Li, J. Liang, S. Xie, Y. Jin, Temperature variation on the central Tibetan Plateau revealed by glycerol dialkyl glycerol tetraethers from the sediment record of Lake Linggo Co since the last deglaciation. *Front. Earth Sci.* **8**, 574206 (2020).

65. J. Hou, Q. Tian, M. Wang, Variable apparent hydrogen isotopic fractionation between sedimentary n-alkanes and precipitation on the Tibetan Plateau. *Org. Geochem.* **122**, 78–86 (2018).

66. X. Huang, R. D. Pancost, J. Xue, Y. Gu, R. P. Evershed, S. Xie, Response of carbon cycle to drier conditions in the mid-Holocene in central China. *Nat. Commun.* **9**, 1369 (2018).

67. A. L. Sessions, L. Zhang, P. V. Welander, D. Doughty, R. E. Summons, D. K. Newman, Identification and quantification of polyfunctionalized hopanoids by high temperature gas chromatography–mass spectrometry. *Org. Geochem.* **56**, 120–130 (2013).

68. J. E. Vonk, T. Tesi, L. Bröder, H. Holmstrand, G. Hugelius, A. Andersson, O. Dudarev, I. Semiletov, Ö. Gustafsson, Distinguishing between old and modern permafrost sources in the northeast Siberian land–shelf system with compound-specific δ^2H analysis. *Cryosphere* **11**, 1879–1895 (2017).

69. C. J. Hein, M. Usman, T. I. Eglinton, N. Haghipour, V. V. Galy, Millennial-scale hydroclimate control of tropical soil carbon storage. *Nature* **581**, 63–66 (2020).

70. G. Soulet, L. C. Skinner, S. R. Beaupré, V. Galy, A note on reporting of reservoir ^{14}C disequilibria and age offsets. *Radiocarbon* **58**, 205–211 (2016).

71. G. Soulet, Methods and codes for reservoir–atmosphere ^{14}C age offset calculations. *Quat. Geochronol.* **29**, 97–103 (2015).

72. T. Varga, A. J. T. Jull, Z. Lisztes-Szabó, M. Molnár, Spatial distribution of ^{14}C in tree leaves from Bali, Indonesia. *Radiocarbon* **62**, 235–242 (2020).

73. E. Schefuß, T. I. Eglinton, C. L. Spencer-Jones, J. Rullkötter, R. De Pol-Holz, H. M. Talbot, P. M. Grootes, R. R. Schneider, Hydrologic control of carbon cycling and aged carbon discharge in the Congo River basin. *Nat. Geosci.* **9**, 687–690 (2016).

74. A. C. Parnell, D. L. Phillips, S. Bearhop, B. X. Semmens, E. J. Ward, J. W. Moore, A. L. Jackson, J. Grey, D. J. Kelly, R. Inger, Bayesian stable isotope mixing models. *Environmetrics* **24**, 387–399 (2013).

75. S.-Y. Yu, H. He, P. Cheng, Z. Hou, Depth heterogeneity of soil organic carbon dynamics in a heavily grazed alpine meadow on the northeastern Tibetan Plateau: A radiocarbon-based approach. *J. Geophys. Res. Biogeosci.* **122**, 1775–1788 (2017).

76. C. Mu, T. Zhang, Q. Wu, X. Zhang, B. Cao, Q. Wang, X. Peng, G. Cheng, Stable carbon isotopes as indicators for permafrost carbon vulnerability in upper reach of Heihe River basin, northwestern China. *Quat. Int.* **321**, 71–77 (2014).

77. T. Zhou, Y. S. Lai, Z. H. Yang, Y. H. Shi, X. R. Luo, L. Liu, P. Yu, G. Chen, L. X. Cao, S. H. Fan, C. J. Cai, J. Sun, S. H. Chen, H. Y. Lu, X. L. Ma, S. D. Li, X. L. Tang, Modelling Soil δ13C across the Tibetan Plateau Using Deep-Learning. *J. Environ. Inform.* **44**, 48–60 (2024).

78. A. K. Gupta, D. M. Anderson, J. T. Overpeck, Abrupt changes in the Asian southwest monsoon during the Holocene and their links to the North Atlantic Ocean. *Nature* **421**, 354–357 (2003).

79. H. J. Jin, X. L. Chang, S. L. Wang, Evolution of permafrost on the Qinghai-Xizang (Tibet) Plateau since the end of the late Pleistocene. *J. Geophys. Res. Earth Surf.* **112**, F02S09 (2007).

80. B. Wild, A. Andersson, L. Bröder, J. Vonk, G. Hugelius, J. W. McClelland, W. Song, P. A. Raymond, Ö. Gustafsson, Rivers across the Siberian Arctic unearth the patterns of carbon release from thawing permafrost. *Proc. Natl. Acad. Sci. U.S.A.* **116**, 10280–10285 (2019).

81. K. M. W. Anthony, S. A. Zimov, G. Grosse, M. C. Jones, P. M. Anthony, F. S. Chapin III, J. C. Finlay, M. C. Mack, S. Davydov, P. Frenzel, S. Frolking, A shift of thermokarst lakes from carbon sources to sinks during the Holocene epoch. *Nature* **511**, 452–456 (2014).

82. H. Liu, J. Liu, J. Hu, Y. Cao, S. Xiao, W. Liu, Systematical $\delta^{13}C$ investigations of TOC in aquatic plants, DIC and dissolved CO_2 in lake water from three Tibetan Plateau lakes. *Ecol. Indic.* **140**, 109060 (2022).

83. J. Lattaud, C. De Jonge, A. Pearson, F. J. Elling, T. I. Eglinton, Microbial lipid signatures in Arctic deltaic sediments—Insights into methane cycling and climate variability. *Org. Geochem.* **157**, 104242 (2021).

84. C. Zhang, C. Zhao, S.-Y. Yu, X. Yang, J. Cheng, X. Zhang, B. Xue, J. Shen, F. Chen, Seasonal imprint of Holocene temperature reconstruction on the Tibetan Plateau. *Earth Sci. Rev.* **226**, 103927 (2022).

85. Q. Sun, G. Chu, M. Liu, M. Xie, S. Li, Y. Ling, X. Wang, L. Shi, G. Jia, H. Lü, Distributions and temperature dependence of branched glycerol dialkyl glycerol tetraethers in recent lacustrine sediments from China and Nepal. *J. Geophys. Res. Biogeosci.* **116**, G01008 (2011).

86. J. Liang, Y. Guo, N. Richter, H. Xie, R. S. Vachula, R. L. Lupien, B. Zhao, M. Wang, Y. Yao, J. Hou, J. Liu, J. M. Russell, Calibration and application of branched GDGTs to Tibetan lake sediments: The influence of temperature on the fall of the Guge Kingdom in Western Tibet, China. *Paleoceanogr. Paleoclimatol.* **37**, e2021PA004393 (2022).

87. M. H. in't Zandt, S. Liebner, C. U. Welte, Roles of thermokarst lakes in a warming world. *Trends Microbiol.* **28**, 769–779 (2020).

88. P. Brohan, J. J. Kennedy, I. Harris, S. F. B. Tett, P. D. Jones, Uncertainty estimates in regional and global observed temperature changes: A new data set from 1850. *J. Geophys. Res. Atmos.* **111**, D12106 (2006).

89. B. V. Gaglioti, D. H. Mann, B. M. Jones, J. W. Pohlman, M. L. Kunz, M. J. Wooller, Radiocarbon age-offsets in an arctic lake reveal the long-term response of permafrost carbon to climate change: Radiocarbon age-offsets. *J. Geophys. Res. Biogeosci.* **119**, 1630–1651 (2014).

90. T. I. Eglinton, V. V. Galy, J. D. Hemingway, X. Feng, H. Bao, T. M. Blattmann, A. F. Dickens, H. Gies, L. Giosan, N. Haghipour, P. Hou, M. Lupker, C. P. McIntyre, D. B. Montluçon, B. Peucker-Ehrenbrink, C. Ponton, E. Schefuß, M. S. Schwab, B. M. Voss, L. Wacker, Y. Wu, M. Zhao, Climate control on terrestrial biospheric carbon turnover. *Proc. Natl. Acad. Sci. U.S.A.* **118**, e2011585118 (2021).

91. L. Curtin, W. J. D'Andrea, N. L. Balascio, S. Shirazi, B. Shapiro, G. A. de Wet, R. S. Bradley, J. Bakke, Sedimentary DNA and molecular evidence for early human occupation of the Faroe Islands. *Commun. Earth Environ.* **2**, 253 (2021).

92. A. Callegaro, D. Battistel, N. M. Kehrwald, F. Matsubara Pereira, T. Kirchgeorg, M. del Carmen Villoslada Hidalgo, B. W. Bird, C. Barbante, Fire, vegetation, and Holocene climate in a southeastern Tibetan lake: A multi-biomarker reconstruction from Paru Co. *Clim. Past* **14**, 1543–1563 (2018).

93. C. M. Gibson, L. E. Chasmer, D. K. Thompson, W. L. Quinton, M. D. Flannigan, D. Olefeldt, Wildfire as a major driver of recent permafrost thaw in boreal peatlands. *Nat. Commun.* **9**, 3041 (2018).

94. B. W. Bird, P. J. Polisar, Y. Lei, L. G. Thompson, T. Yao, B. P. Finney, D. J. Bain, D. P. Pompeani, B. A. Steinman, A Tibetan lake sediment record of Holocene Indian summer monsoon variability. *Earth Planet. Sci. Lett.* **399**, 92–102 (2014).

95. D. Fleitmann, S. J. Burns, M. Mudelsee, U. Neff, J. Kramers, A. Mangini, A. Matter, Holocene forcing of the indian monsoon recorded in a stalagmite from Southern Oman. *Science* **300**, 1737–1739 (2003).

96. H. Cheng, R. L. Edwards, A. Sinha, C. Spötl, L. Yi, S. Chen, M. Kelly, G. Kathayat, X. Wang, X. Li, X. Kong, Y. Wang, Y. Ning, H. Zhang, The Asian monsoon over the past 640,000 years and ice age terminations. *Nature* **534**, 640–646 (2016).

97. P. O. Hopcroft, P. J. Valdes, D. J. Beerling, Simulating idealized Dansgaard-Oeschger events and their potential impacts on the global methane cycle. *Quat. Sci. Rev.* **30**, 3258–3268 (2011).

98. B. Ringeval, P. O. Hopcroft, P. J. Valdes, P. Ciais, G. Ramstein, A. J. Dolman, M. Kageyama, Response of methane emissions from wetlands to the Last Glacial Maximum and an idealized Dansgaard–Oeschger climate event: Insights from two models of different complexity. *Clim. Past* **9**, 149–171 (2013).

99. Y. Zheng, Z. Fang, T. Fan, Z. Liu, Z. Wang, Q. Li, R. D. Pancost, B. D. A. Naafs, Operation of the boreal peatland methane cycle across the past 16 k.y. *Geology* **48**, 82–86 (2020).

100. Y. Zheng, J. S. Singarayer, P. Cheng, X. Yu, Z. Liu, P. J. Valdes, R. D. Pancost, Holocene variations in peatland methane cycling associated with the Asian summer monsoon system. *Nat. Commun.* **5**, 4631 (2014).

101. M. Elvert, J. W. Pohlman, K. W. Becker, B. Gaglioti, K.-U. Hinrichs, M. J. Wooller, Methane turnover and environmental change from Holocene lipid biomarker records in a thermokarst lake in Arctic Alaska. *Holocene* **26**, 1766–1777 (2016).

102. B. Aichner, H. Wilkes, U. Herzschuh, S. Mischke, C. Zhang, Biomarker and compound-specific $\delta^{13}C$ evidence for changing environmental conditions and carbon limitation at Lake Koucha, eastern Tibetan Plateau. *J. Paleolimnol.* **43**, 873–899 (2010).

103. K. M. W. Anthony, P. Lindgren, P. Hanke, M. Engram, P. Anthony, R. P. Daanen, A. Bondurant, A. K. Liljedahl, J. Lenz, G. Grosse, B. M. Jones, L. Brosius, S. R. James, B. J. Minsley, N. J. Pastick, J. Munk, J. P. Chanton, C. E. Miller, F. J. Meyer, Decadal-scale hotspot methane ebullition within lakes following abrupt permafrost thaw. *Environ. Res. Lett.* **16**, 035010 (2021).

104. E. Fluet-Chouinard, B. D. Stocker, Z. Zhang, A. Malhotra, J. R. Melton, B. Poulter, J. O. Kaplan, K. K. Goldewijk, S. Siebert, T. Minayeva, G. Hugelius, H. Joosten, A. Barthelmes, C. Prigent, F. Aires, A. M. Hoyt, N. Davidson, C. M. Finlayson, B. Lehner, R. B. Jackson, P. B. McIntyre, Extensive global wetland loss over the past three centuries. *Nature* **614**, 281–286 (2023).

105. Y. Xi, S. Peng, A. Ducharne, P. Ciais, T. Gumbricht, C. Jimenez, B. Poulter, C. Prigent, C. Qiu, M. Saunois, Z. Zhang, Gridded maps of wetlands dynamics over mid-low latitudes for 1980–2020 based on TOPMODEL. *Sci. Data* **9**, 347 (2022).

106. X. Pi, Q. Luo, L. Feng, Y. Xu, J. Tang, X. Liang, E. Ma, R. Cheng, R. Fensholt, M. Brandt, X. Cai, L. Gibson, J. Liu, C. Zheng, W. Li, B. A. Bryan, Mapping global lake dynamics reveals the emerging roles of small lakes. *Nat. Commun.* **13**, 5777 (2022).

107. L. Foster, E. Pearson, S. Juggins, D. Hodgson, K. Saunders, E. Verleyen, S. Roberts, Development of a regional glycerol dialkyl glycerol tetraether (GDGT)–temperature calibration for Antarctic and sub-Antarctic lakes. *Earth Planet. Sci. Lett.* **433**, 370–379 (2015).

108. J. H. Raberg, D. J. Harning, S. E. Crump, G. de Wet, A. Blumm, S. Kopf, Á. Geirsdóttir, G. H. Miller, J. Sepúlveda, Revised fractional abundances and warm-season temperatures substantially improve brGDGT calibrations in lake sediments. *Biogeosciences* **18**, 3579–3603 (2021).

109. C. De Jonge, E. C. Hopmans, C. I. Zell, J.-H. Kim, S. Schouten, J. S. Sinninghe Damsté, Occurrence and abundance of 6-methyl branched glycerol dialkyl glycerol tetraethers in soils: Implications for palaeoclimate reconstruction. *Geochim. Cosmochim. Acta* **141**, 97–112 (2014).

110. S. Kusch, M. Winterfeld, G. Mollenhauer, S. T. Höfle, L. Schirrmeister, G. Schwamborn, J. Rethemeyer, Glycerol dialkyl glycerol tetraethers (GDGTs) in high latitude Siberian permafrost: Diversity, environmental controls, and implications for proxy applications. *Org. Geochem.* **136**, 103888 (2019).

111. C. A. Dykoski, R. L. Edwards, H. Cheng, D. Yuan, Y. Cai, M. Zhang, Y. Lin, J. Qing, Z. An, J. Revenaugh, A high-resolution, absolute-dated Holocene and deglacial Asian monsoon record from Dongge Cave, China. *Earth Planet. Sci. Lett.* **233**, 71–86 (2005).

112. Y. Tian, D. Fleitmann, Q. Zhang, L. Sha, J. A. Wassenburg, J. Axelsson, H. Zhang, X. Li, J. Hu, H. Li, L. Zhao, Y. Cai, Y. Ning, H. Cheng, Holocene climate change in southern Oman deciphered by speleothem records and climate model simulations. *Nat. Commun.* **14**, 4718 (2023).

113. G. Kathayat, H. Cheng, A. Sinha, L. Yi, X. Li, H. Zhang, H. Li, Y. Ning, R. L. Edwards, The Indian monsoon variability and civilization changes in the Indian subcontinent. *Sci. Adv.* **3**, e1701296 (2017).

114. J. Laskar, P. Robutel, F. Joutel, M. Gastineau, A. C. M. Correia, B. Levrard, A long-term numerical solution for the insolation quantities of the Earth. *Astron. Astrophys.* **428**, 261–285 (2004).
